# An M/M/c/K State-Dependent Model for Pedestrian Flow Control and Design of Facilities

**DOI:** 10.1371/journal.pone.0133229

**Published:** 2015-07-21

**Authors:** Khalidur Rahman, Noraida Abdul Ghani, Anton Abdulbasah Kamil, Adli Mustafa, Md. Ahmed Kabir Chowdhury

**Affiliations:** 1 Department of Statistics, School of Physical Sciences, Shahjalal University of Science and Technology, Sylhet, 3114, Bangladesh; 2 Mathematics Section, School of Distance Education, Universiti Sains Malaysia, 11800, USM, Penang, Malaysia; 3 School of Mathematical Sciences, Universiti Sains Malaysia, 11800, USM, Penang, Malaysia; Universidad Veracruzana, MEXICO

## Abstract

Pedestrian overflow causes queuing delay and in turn, is controlled by the capacity of a facility. Flow control or blocking control takes action to avoid queues from building up to extreme values. Thus, in this paper, the problem of pedestrian flow control in open outdoor walking facilities in equilibrium condition is investigated using M/M/c/K queuing models. State dependent service rate based on speed and density relationship is utilized. The effective rate of the Poisson arrival process to the facility is determined so as there is no overflow of pedestrians. In addition, the use of the state dependent queuing models to the design of the facilities and the effect of pedestrian personal capacity on the design and the traffic congestion are discussed. The study does not validate the sustainability of adaptation of Western design codes for the pedestrian facilities in the countries like Bangladesh.

## Introduction

Flow control is one of the common means to ensure proper quality of service with limited facilities in transportation and communication industry. An efficient flow control approach is the one that adjusts the input flow based on the appropriate operating characteristics to provide optimum performances. Therefore, the real factors that have significant influences on the operating characteristics and the performances should be incorporated as much as possible in the flow control approach. The incorporation and the assessment of influences are usually done by using an analytical model. However, the opportunity to find an appropriate analytical tool is very limited as few real-world situations conform to the requisite assumptions. This causes it difficult for operators to sharply respond to the changes in traffic conditions and for planners to provide effective design for the capacity of the facilities in transportation and communication industry.

The pedestrian mode is a transportation mode that enables people to travel the areas where vehicles are restricted to access or are not desired for safety and environmental motivation. In addition, the utilization of such transportation mode lessens the use of energy resources, and increases physical fitness of people. The Central Business District (CBD) of a city is ideally suited for the provision of efficient pedestrian networks, although in such area the intermixing of pedestrians and vehicle traffic is a common phenomenon. Thus, in addition to setting up new pedestrian facilities, pedestrian flows should be properly controlled to enhance walking environment, mitigate environmental pollution, and reduce the number of victims due to traffic fatalities in the CBD. The pedestrian facilities in CBD areas should not turn into bottlenecks/ high congestions. On the other hand, although the increment of capacity (additional line) eases the pedestrian jam, the necessity of additional line on the valuable land in CBD should be justified against the monetary cost and based on how the users rank delays due to congestion [1]. Braess[2] noticed that, in the congested traffic condition, addition of line to the capacity leads to an increase in the average travel time for everyone. This is because self-serving individuals cannot refrain themselves from utilizing the additional capacity, even though such utilization leads to deterioration in the average travel time. Thus, one way to avoid the bottlenecks is to control the flow based on the local pedestrian flow characteristics [3,4] and to design the pedestrian facilities according to their demand for flow. The pedestrian flows and facilities should also be analysed with an appropriate analytical tool that can incorporate important determinants and functional factors. However, there are a small number of researches that have devoted to control the pedestrian flow as well as the capacity based design of pedestrian facilities.Hu, Jiang, Zhu, & Chen [5] has established the quantitative relationship between LOS and metro station corridor width based on PH/PH(n)/*C*/*C*, where, PH denotes the phase-type distribution.Yuhaski& Smith [6] and Mitchell & MacGregor Smith [7] presented comprehensive analyses of pedestrian facilities and developed an analytical approximation methodology based on M/G/C/C state dependent queuing models to compute certain performance measures for pedestrian flows and the design of facilities. However, to overcome some limitations and to incorporate some important determinants and functional factors, as mentioned in the next section and thereafter, in this study we will use the M/M/c/K state-dependent queuing models to control pedestrian flows and capacity based design of pedestrian facilities in open outdoor walking environment e.g. sidewalks. Thus the main novelty of the proposed methodology is to make the use of realistic relationships among pedestrian traffic flow variables to control pedestrian flows and the design of sidewalk facilities. The studies on pedestrian movements can be categorized as microscopic and macroscopic models [4]. Microscopic models include agent-based [8], cellular based [9], and lattice gas model based [10] simulation models. Queuing theory based models can be viewed as macroscopic models[11].

The rest of the paper is organized in the following manner. The reasons for using M/M/c/K state dependent queuing models and the pedestrian speed model that can represent the state dependent services on sidewalks are presented in Section 2. The background to the use and development of the M/M/c/K state-dependent pedestrian flow control model to analyze a single link of a network of sidewalks is provided in Section 3, which could be extended to analyse pedestrian flows through the network. Section 4 presents some sensitivity analysis to show how the developed M/M/c/K state-dependent flow control model can be used in avoiding bottlenecks and in the planning and design/sizing of pedestrian sidewalk facilities. The paper ends with conclusions.

## Queuing and Congestion Models for Sidewalks

Crowd on the sidewalks in a CBD sometimes brings the pedestrian movements to a standstill. In such situations, a pedestrian could not freshly join the queue on the facility because the facility is already at the capacity (i.e. balk). Balking is not only a common phenomenon in the pedestrian movements, but it is also frequently observed in vehicle traffic, machine repair models etc. Haight [12] initialized the concept of balking for an M/M/1 queue. The time-dependent solution for an M/M/1 queue with balking has been provided by Kumar, Parthasarathy, &Sharafali[13]. An extension of the M/M/1 queuing process with a spatial structure and excluded-volume effect has been introduced by Arita[14]. In 1917 Danish mathematician A K Erlang gave a formula for loss and waiting time based on M/M/s/s system, which was soon used by many telephone companies in different countries [15,16]. The loss formula is known as Erlang’s B formula, where B stands for blocking, and can be used for estimating the probability of balking in telephone, cable etc. This loss formula has also been used by Yuhaski& Smith [6] and Mitchell & MacGregor Smith [7] in the performance measures and the planning of pedestrian facilities and networks. However, the following reasons have stimulated to adopt the M/M/c/K system based queuing models rather than M/G/C/C queuing models for the design and analysis of pedestrian networks in open outdoor walking facilities:

1. The M/G/C/C state dependent queuing models consider that the queue consists entirely of the walking facility without any buffer space. Such consideration may be applicable in an emergency evacuation from a building or in circuit switching. However, it is not reasonable for the uninterrupted and moving pedestrians in an open outdoor walking facility/ sidewalk.
2. The waiting/lingering time, and the lateral spacing required for the movements of a pedestrian on a walking facility are not explicitly reflected in the formulation of the corresponding congestion models.
3. The congestion models for M/G/C/C state-dependent queuing models do not support the observation of Polus, Schofer, & Ushpiz[17], that is to say, up to the densities of about 0.6 ped./m^2^ the free flow condition remains valid on sidewalk facilities.

The M/M/s system based congestion models as derived in Eq 1 based on the model developed by Rahman et al. [18] (which has been empirically validated based on the data on the pedestrian characteristics on some sidewalks in Dhaka, Bangladesh), and M/M/c/K state-dependent pedestrian flow models, as formulated in Section 3, could triumph over these shortcomings to study the pedestrian movements and flow control on sidewalk facilities, and designing of such facilities.

### 2.1 Pedestrian Walking Speeds on Sidewalks

Many studies on pedestrian movements have been carried out under different conditions. The studies found that the patterns of pedestrian movements under the normal and emergency conditions are not same. However, there are some common personal factors such as age, gender, intelligence, and physical fitness of a pedestrian have significant influence on the pedestrian speeds in any walking condition and environment. Since pedestrian free flow speed is mainly influenced by the pedestrian variables or personal attributes, the inclusion of most common factors to the modelling of pedestrian movements can be done by bringing free flow speed to the corresponding congestion models.

In a public walkway facility, the usual movements of a pedestrian are hindered by the presence of other pedestrians [19]. Thus, on the sidewalks in a CBD, interaction with other pedestrians is the most important factor that influences the pedestrian speed and flow. People moving on the sidewalks are not always in huge numbers, but certainly at high densities. On the sidewalks, pedestrians usually travel at a maximum density of 1.55 ped./m^2^ (normal capacity), whereas pedestrian free flow speeds start to decline at a density of 0.6 ped./m^2^ and ‘usual jam’ (the facility is at the capacity) occurs at densities of about 3.32 ped./m^2^ [4,17,18]. As far as continuous pedestrian movements on sidewalks is concerned, ‘usual jam’ leading to ‘solid jamming’ seemingly takes place at densities in the range 4 to 5 ped./m^2^, which is very rare to be occurred. Rahman et al. [18] have developed a non-linear analytical model for pedestrian speeds on sidewalks, which supports the above mentioned empirical results and can be expressed as Eq 1 as a function of the number of pedestrians on a sidewalk.

### 2.2 Congestion Model for Sidewalks

**Fig 1:**
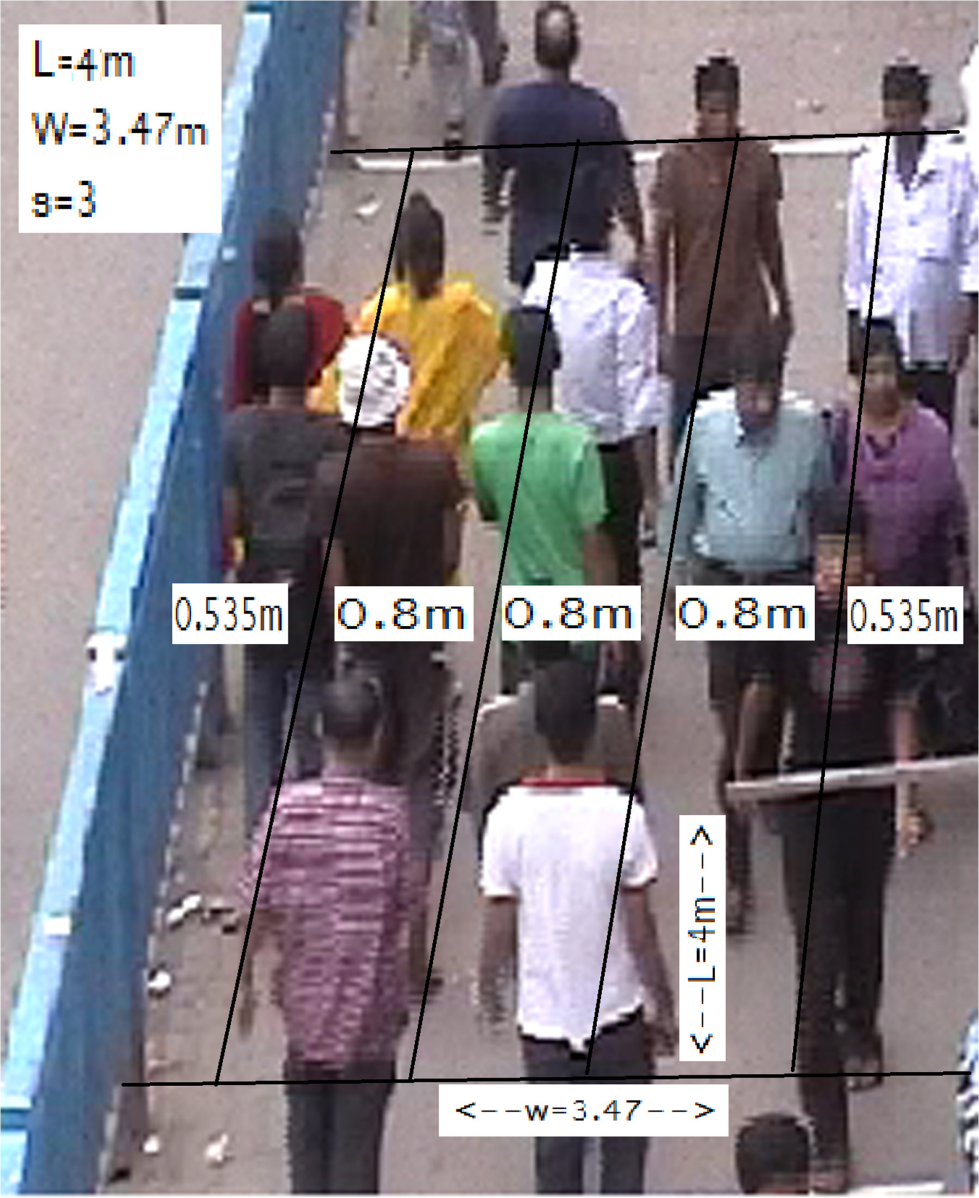
Illustration of the Congestion Model on a 4 m × 3.47 m Sidewalk.

In a congested situation, the three variables that completely describe the pedestrian traffic flows are speed, flow and density. Pedestrian flows moving on a facility represent pedestrians’ demand. Depending on the volume of flow and other factors (e.g. personal attributes) speed and density will fluctuate. Confronting the demand for flow and the capacity of the facility (supply) determines the operating characteristics and the performances of a pedestrian infrastructure under investigation. Speed is a key measure to the quality of service (service rate) provided to the pedestrians on the facility and as such determines the effectiveness of the facility infrastructure [20]. In addition to personal attributes, the average speed of pedestrians is influenced by many other factors including the purpose of the journey, the physical nature of the walkway, the nature of the surrounding area, and weather [21]. However, for the purpose of capacity analysis only concentration (the number of pedestrians in the facility) should be considered [22]. Hence, the speed-density relationship on sidewalks developed by Rahman et al. [18] has been adopted in this study and it can be expressed as the following as a function of the number of pedestrians on a sidewalk (Fig 1).

$$v_{m}=\frac{v_{f}}{1+\frac{{\left(\frac{m}{c}\right)}^{s}}{s\left(1-\frac{m}{s*c}\right){\left(\frac{m}{c}\right)}^{s}+s(s!){\left(1-\frac{m}{s*c}\right)}^{2}\sum_{n=0}^{s-1}\left(\frac{{\left(\frac{m}{c}\right)}^{n}}{n!}\right)}}$$ (1)

where

*v*_*m*_ = average walking speed of *m* pedestrians on the facility (m/sec);

*v*_*f*_ = average free flow speed of a pedestrian (m/sec);

*c* = *1*.55*W*L = the normal capacity of a sidewalk facility (ped.);

*W* = the width of the facility (m); *L* = the length of the facility (m);

*m* = number of pedestrians on the facility, *m* = 1, 2, …,*c*,…,K (= 2*c*);

*s* = $\frac{W-1.07}{b}$ = number of pedestrian lanes on the facility, where 1.07 m of width is reduced to calculate the effective width of the facility [22] and

*b* = lateral spacing required for a pedestrian to move on the facility (m) = 0.8m [20].

With the help of the above congestion model one can calculate the different speed rates for the variation of the number of pedestrians on a sidewalk. The model supports the observation of Polus et al. [17]. In other words, it follows that the pedestrian can move without any obstacle up to the densities of about 0.6 ped./m^2^. As mentioned in sub-section 2.1, pedestrians on sidewalks usually move at maximum density of 1.55 ped./m^2^. Therefore, the normal capacity, *c*, is equal to 1.55 times the area of the facility in square meters (m^2^). Hence, in the above model, *c* is expressed as 1.55*W*L. In addition, the use of maximum density of 1.55 ped./m^2^ in the model estimates that the ‘usual jam’ (the facility is at the capacity) occurs at densities of about 3.32 ped./m^2^. Thus, it is reasonable to consider that the facility will be at jam capacity when there are more 2c pedestrians on the facility. Therefore, in the above model, it is considered that the highest number of pedestrians on the facility could be up to K = 2*c*. For illustration and experimentation purposes, we will be confined ourselves to the above expression and consideration. It should be noted that the considered model does not take into account the ‘solid jam’ condition of 4 to 6 ped./m^2^, which is usually occurred in an emergency evacuation from a building or a stadium.

## Formulation of Analytical Model for a Single Sidewalk

It is mentioned in the sub-section 2.2 that pedestrian speed, flow and density (the number of pedestrians on a facility) completely describe the pedestrian traffic flows in a congested situation. Thus, it is always favourable if the performance measures of a pedestrian facility could be formulated based on these three variables. In the study of stochastic nature of pedestrian flows, when two of the three variables are known, the traffic operator can control the third one to meet performance measures to the targeted magnitudes. Based on the capacity of a facility, the pedestrian flow ascertains the operating characteristics and the performances of an infrastructure under investigation. Thus, the controlling of pedestrian flows for given speeds and the number of pedestrians on a facility is more convenient in terms of sharply response to the stochastic changes in pedestrian traffic conditions.

In the development of analytical models for understanding the stochastic nature of pedestrian flows, we can define a single sidewalk as a station where pedestrians are served. It is considered that the sidewalk has *c* servers in the normal capacity (as discussed in the previous section), which provide facilities to the pedestrians to pass the sidewalk without overflowed. A pedestrian can be both an output and an input to the queuing system within the sidewalk. Here, we assume that pedestrians enter the sidewalk in accordance with a Poisson process with rate *λ*(and thus the inter-entrance times are exponentially distributed), enter the sidewalk if it is not in jam capacity K, and then spend an exponential amount of time on the sidewalk with rate *μ*_*m*_ being served. The service time is equal to the travel time required for a pedestrian to pass the entire length of the sidewalk. The travel time and hence the service rate, *μ*_*m*_, is state dependent as the travel time of each pedestrian within the sidewalk depends on the number of prevailing pedestrians on the sidewalk. It is assumed that, at each moment of time, pedestrians are uniformly distributed over the sidewalk. Thus, for *m* pedestrians on the sidewalk, the service rate will be a function of *m* i.e. *f*(*m*). Since the sidewalk normal capacity is *c* and jam capacity is K, we can model the stochastic nature of pedestrian flows on a sidewalk with a queuing model. Thus, our considered model, in Kendall notation, could be described as M/M/c/K. General concepts of Kendall notation are described in the Appendix.

**Fig 2:**
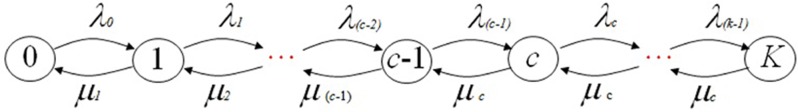
State-transition-rate Diagram.

From the state-transition-rate diagram (Fig 2) and the balance equations for M/M/c/K queuing system, we have the steady-state probabilities *p*_*m*_ (m = 1, 2…,c,…K) of *m* pedestrians on the sidewalk as

$$p_{m}=\{\begin{array}{c} \frac{\lambda_{0}\lambda_{1}\ldots \ldots \ldots \ldots \lambda_{m-1}}{\mu_{1}\mu_{2}\ldots \ldots \ldots ..\mu_{m}}p_{0}\;for\;1\leq m\leq c\\ \frac{\lambda_{0}\lambda_{1}\ldots \ldots \ldots \ldots \lambda_{m-1}}{\mu_{1}\mu_{2}\ldots ..\mu_{c}{\left(\mu_{c}\right)}^{m-c}}p_{0}\;for\;c\leq m\leq K \end{array}$$ (2)

From the normalizing condition for *p*_0,_ probability of no pedestrian on the sidewalk, we have

$$p_{0}={\left(1+\sum_{m=1}^{c-1}\frac{\lambda_{0}\lambda_{1}\ldots \ldots \ldots \ldots \lambda_{m-1}}{\mu_{1}\mu_{2}\ldots \ldots \ldots ..\mu_{m}}+\sum_{m=c}^{K}\frac{\lambda_{0}\lambda_{1}\ldots \ldots \ldots \ldots \lambda_{m-1}}{\mu_{1}\mu_{2}\ldots ..\mu_{c}{\left(\mu_{c}\right)}^{m-c}}\right)}^{-1}$$ (3)

In the flow control modelling, we consider that the arrival rates will be controlled (for example, by using a roundabout on the middle or in the entry and exit points of the sidewalk) and hence arrival rates are not influenced by the number of prevailing pedestrians, *m*, on the sidewalk. We, therefore, assume that the arrival rates of flows are constant such that *λ* = *λ*_0_ = *λ*_1_ = ……. = *λ*_*c*_ = ….. = *λ*_*K*−1_ and then we have from Eqs 2 and 3

$$p_{m}=\{\begin{array}{c} \frac{\lambda^{m}}{\prod_{i=1}^{m}\mu_{i}}p_{0}\;for\;1\leq m\leq c\\ \frac{\lambda^{m}}{{\left(\mu_{c}\right)}^{m-c}\prod_{i=1}^{m}\mu_{i}}p_{0}\;for\;c\leq m\leq K \end{array}$$ (4)

and

$$p_{0}={\left(1+\sum_{m=1}^{c-1}\frac{\lambda^{m}}{\prod_{i=1}^{m}\mu_{i}}+\sum_{m=c}^{K}\frac{\lambda^{m}}{{\left(\mu_{c}\right)}^{m-c}\prod_{i=1}^{m}\mu_{i}}\right)}^{-1}$$ (5)

where *μ*_*i*_, for i = 1, 2…..*m*, is a function of i, the number of pedestrians on the sidewalk. Since the number of prevailing pedestrians affects the average pedestrian speed/travel time and hence the service rates, we can use the non-linear congestion model of Eq 1 to describe *μ*_*m*_ on a single sidewalk. Note that the service rate, *r*_m,_ when there are *m* pedestrians on the sidewalk, is equal to the inverse of the average time that is required for a pedestrian to traverse the length of the sidewalk; therefore,

$$r_{m}=\frac{v_{m}}{L}$$ (6)

Since, in our consideration, *m* servers simultaneously serve on the sidewalk facility, we will have the overall service rate for *m* pedestrians as

$$\mu_{m}=mr_{m}=m\frac{v_{m}}{L}$$ (7)

By substituting the expression for *μ*_*m*_, from Eq 7 into Eqs 4 and 5, we obtain the steady-state probabilities as

$$p_{m}=\{\begin{array}{c} \frac{{\left[\lambda E(S)\right]}^{m}}{m!\prod_{i=1}^{m}f(i)}p_{0}\;for\;1\leq m\leq c\\ \frac{{\left[\lambda E(S)\right]}^{m}}{c!{\left(c\right)}^{m-c}\prod_{i=1}^{m}f(i)}p_{0}\;for\;c\leq m\leq K \end{array}$$ (8)

and

$$p_{0}={\left(1+\sum_{m=1}^{c-1}\frac{{\left[\lambda E(S)\right]}^{m}}{m!\prod_{i=1}^{m}f(i)}+\sum_{m=c}^{K}\frac{{\left[\lambda E(S)\right]}^{m}}{c!{\left(c\right)}^{m-c}\prod_{i=1}^{m}f(i)}\right)}^{-1}$$ (9)

where $E\left(S\right)=\frac{L}{v_{f}}$ is the expected service time/ traverse time for a pedestrian in free flow condition in a sidewalk of length L and

$$f(m)=\frac{v_{m}}{v_{f}}={\left[1+\frac{{\left(\frac{m}{c}\right)}^{s}}{s\left(1-\frac{m}{s*c}\right){\left(\frac{m}{c}\right)}^{s}+s(s!){\left(1-\frac{m}{s*c}\right)}^{2}\sum_{n=0}^{s-1}\left(\frac{{\left(\frac{m}{c}\right)}^{n}}{n!}\right)}\right]}^{-1}$$

is the ratio of average speed of *m* pedestrians on the sidewalk to that of speed in free flow condition. Since the speed has been expressed as a function of number of pedestrians or density, for a particular number of pedestrians on a given facility, the steady-state probabilities and the corresponding performance measures (as will be discussed in the next section) will depend on the arrival rates i.e. on the pedestrian flows.

## Experiments with the Model and Optimization

In this section a sensitivity analysis is performed based on the developed congestion model in Eq 1 to examine the effect of different factors on pedestrian flows and performances. The use of M/M/c/K queuing models for analysing a single sidewalk allows us to compute certain performance measures in equilibrium condition. The most relevant performance measures include

1. The probability of balking (*p*_*Balk*_) is equal to *p*_*m*_ where m equals K,
2. $E(Q)=\sum_{m=c+1}^{K}(m-c)p_{m}$ is the average number of pedestrians waiting in the queue in the equilibrium condition,
3. $E(T)=\frac{\sum_{m=1}^{K}mp_{m}}{\theta }$ is the expected amount of time a pedestrian spends on the facility,
4. *θ* = *λ*(1 − *p*_*Balk*_) is the pedestrian effective arrival to the sidewalk or throughput through the sidewalk.

The most important factors that influencing the behaviour of pedestrian flows and the related performances of a walking facility include pedestrian arrival flow rate, *λ*, length of the facility L, width of the facility W and pedestrian personal capacity *v*_*f*_. The effects of these factors will be examined in the current study. The examination of first factor is necessary to determine the effect of arrival rate of pedestrians on flows and to determine the effective arrival rate so that there is no overflow of pedestrians on the facility. The identification of optimal values for the second and third factors is helpful for the proper sizing and design of pedestrian facilities. The inspection of last factor shows how pedestrian personal capacity influences the traffic congestion on a walking facility and it also helps in the sizing of a facility. For a given set of values for pedestrian arrival flow rate, average free flow speed, length and width of a facility, a particular set of performances could be observed for the facility. However, by fixing up three of these four factors constant, the effect of the remaining factor on the performances can be analysed. Thus, in the following subsections, the effects of pedestrian arrival flow rate, width and length of the facility and pedestrian personal capacity on the selected performances are studied, respectively.

### 4.1 The Effect of Pedestrian Arrival Rate on Performances

To determine the effective arrival rate of pedestrians so that there is no overflow of pedestrians on the facility and optimal performances are achieved, we could fix the length, width of the facility, and the pedestrian free flow speed capacity. Then we can vary the arrival rate of pedestrians to the facility/ sidewalk to find out a most favourable arrival rate. In Table 1 different performances of an 8 m by 3 m (length by width) sidewalk for pedestrians in Dhaka, Bangladesh are presented. Rahman et al. [3] reported that average free flow speed of the pedestrians in Dhaka is 1.20 m/sec. Different arbitrary arrival rates (in ped./sec) are provided in the first column. For each arrival rate, in equilibrium condition, the probability of balking and the average number of pedestrians (ped.) in the queue inside the facility are computed in the second and third columns, respectively. In the fourth and last columns, expected amount of time (in sec) that a pedestrian needs to pass the facility and the pedestrian throughput through (ped./sec) the facility are calculated, correspondingly. The table shows that as the arrival rate increases from 3.5 to 4 (ped./sec), the number of pedestrians in the queue inside the sidewalk increases from around 0 to 25 (ped.). There is no congestion inside the sidewalk for the arrival rates lower than or equal to 3.5 (ped./sec). In this interval of arrival rates, each probability of balking is approximately equal to 0, which results in that the effective arrival rates or throughputs equal to actual arrival rates. At non-congestion conditions, the average time that that a pedestrian needs to pass the facility is approximately equal to the result of the length (8 m) divided by pedestrian free flow speed capacity (1.20 m/sec).

**Table 1:** Performances of 8 m × 3 m sidewalk for different arrival rates with *v*_*f*_ = 1.2 m/sec.

Arrival Rate	Balking Probability	Pedestrians in Queue	Average time for a pedestrian	Throughput
(*λ*)	(*p*_*Balk*_)	*E*(*Q*)	*E*(*T*)	(*θ*)
1	0.00000	0.00000	6.68913	1.00000
2	0.00000	0.00000	6.76306	2.00000
3	0.00000	0.00183	6.91780	2.99999
3.5	0.00137	0.19077	7.12444	3.49519
3.65	0.01121	1.18176	7.60415	3.60908
3.7	0.02147	2.14274	8.04274	3.62055
3.75	0.03966	3.78526	8.79399	3.60129
3.8	0.06968	6.41150	10.03149	3.53522
3.9	0.17340	15.05816	14.64081	3.22373
4	0.30306	25.16002	21.64213	2.78775
5	0.56872	37.16916	34.85847	2.15641
6	0.64517	37.42101	35.42542	2.12901
7	0.69791	37.55126	35.72795	2.11463
8	0.73680	37.63275	35.92021	2.10558
9	0.76674	37.68888	36.05394	2.09932

**Fig 3:**
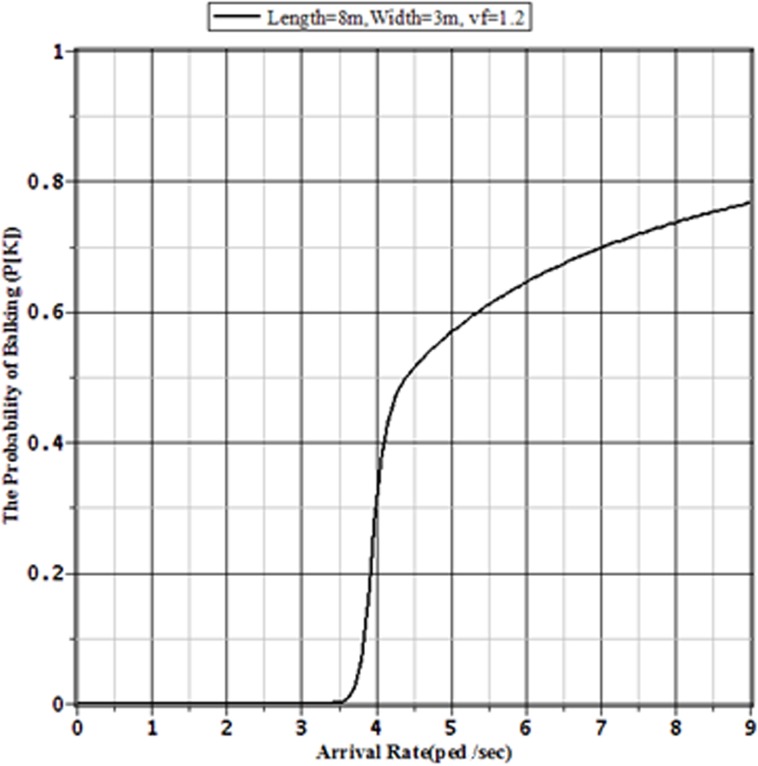
Probabilities of Balking for Different Arrival Rates to an 8 m × 3 m Sidewalk with v_*f*_ = 1.2 m/sec.

**Fig 4:**
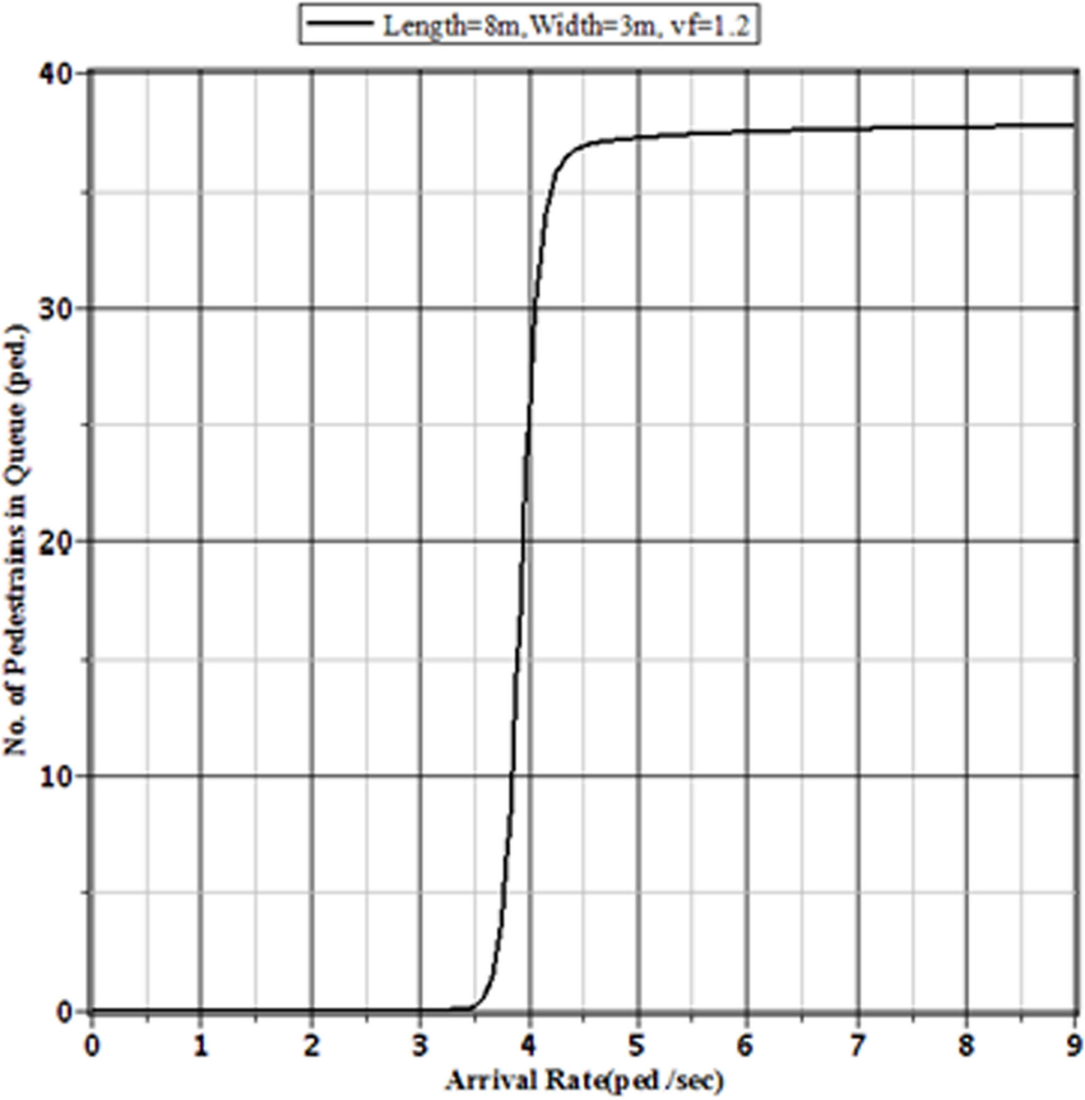
Expected Number of Pedestrians in Queue on the Facility for Different Arrival Rates to an 8 m × 3 m Sidewalk with v_*f*_ = 1.2 m/sec.

**Fig 5:**
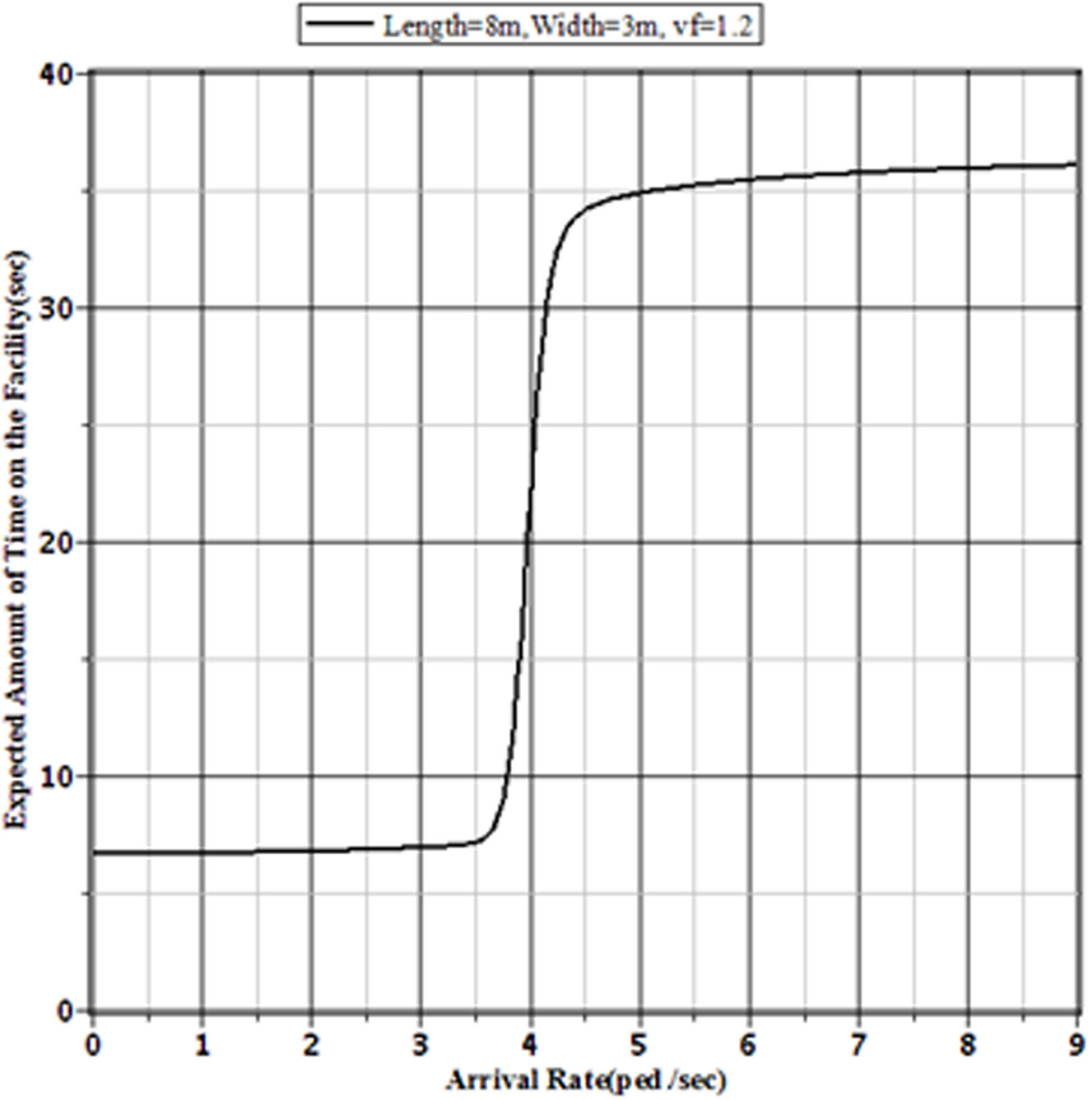
Expected Service Times for Different Arrival Rates to an 8 m × 3 m Sidewalk with v_*f*_ = 1.2 m/sec.

**Fig 6:**
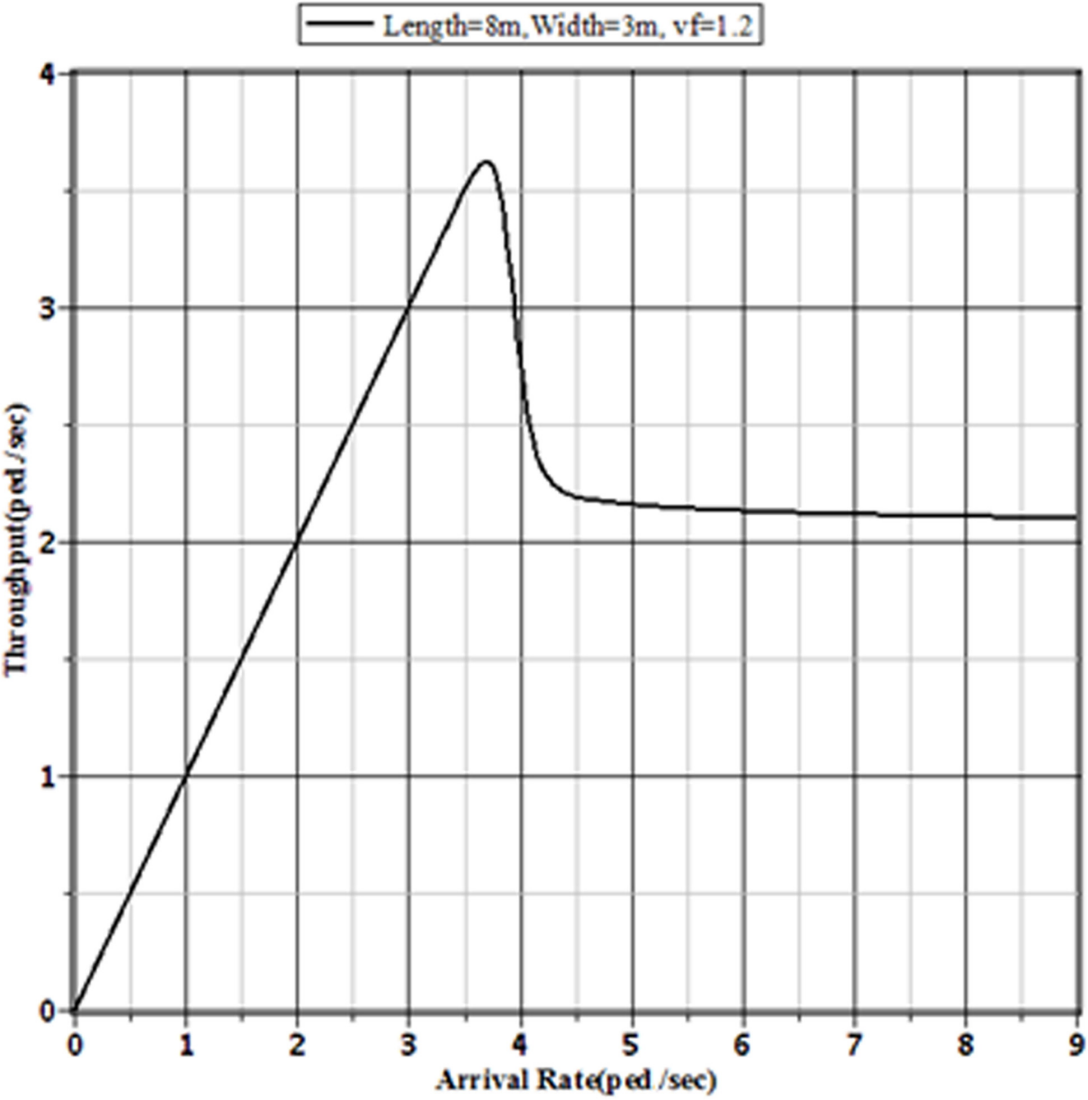
Throughputs of the Facility for Different Arrival Rates to an 8 m × 3 m Sidewalk with v_*f*_ = 1.2 m/sec.

Fig 3, Fig 4, Fig 5 and Fig 6 show the probabilities of balking, the average number of pedestrians waiting in the queue, the expected amount of time a pedestrian spends on the facility and pedestrian effective arrival to the sidewalk or throughput through the sidewalk, respectively, for the selected sidewalk of 8 m × 3 m at different arrival rates. Fig 6 shows that the throughput is maximized when the pedestrian arrival rate reaches the critical value at around 3.7 ped./sec. Beyond this arrival rate, the throughput starts to decrease with the increment of arrival rate. This is happened for the faster increment of probability of balking beyond the critical value of arrival rate. The number of pedestrians in the queue and the expected time needed to pass the sidewalk by a pedestrian both increase beyond the critical value. As the number of pedestrians in the sidewalk increases, the sidewalk capacity moves towards the usual jam capacity. In such situation, there is a little effect of the increment of arrival rate on the pedestrian throughput. Hence, the pedestrian throughput converges to a limit value. To avoid the building of extreme queue in the sidewalk facilities, the traffic operators should control the pedestrian flow before reaching the arrival rate to the limit value. For a sidewalk of 8 m × 3 m in Dhaka, this limit value of pedestrian arrival is somewhere between 4.5 to 5 ped./sec.

**Fig 7:**
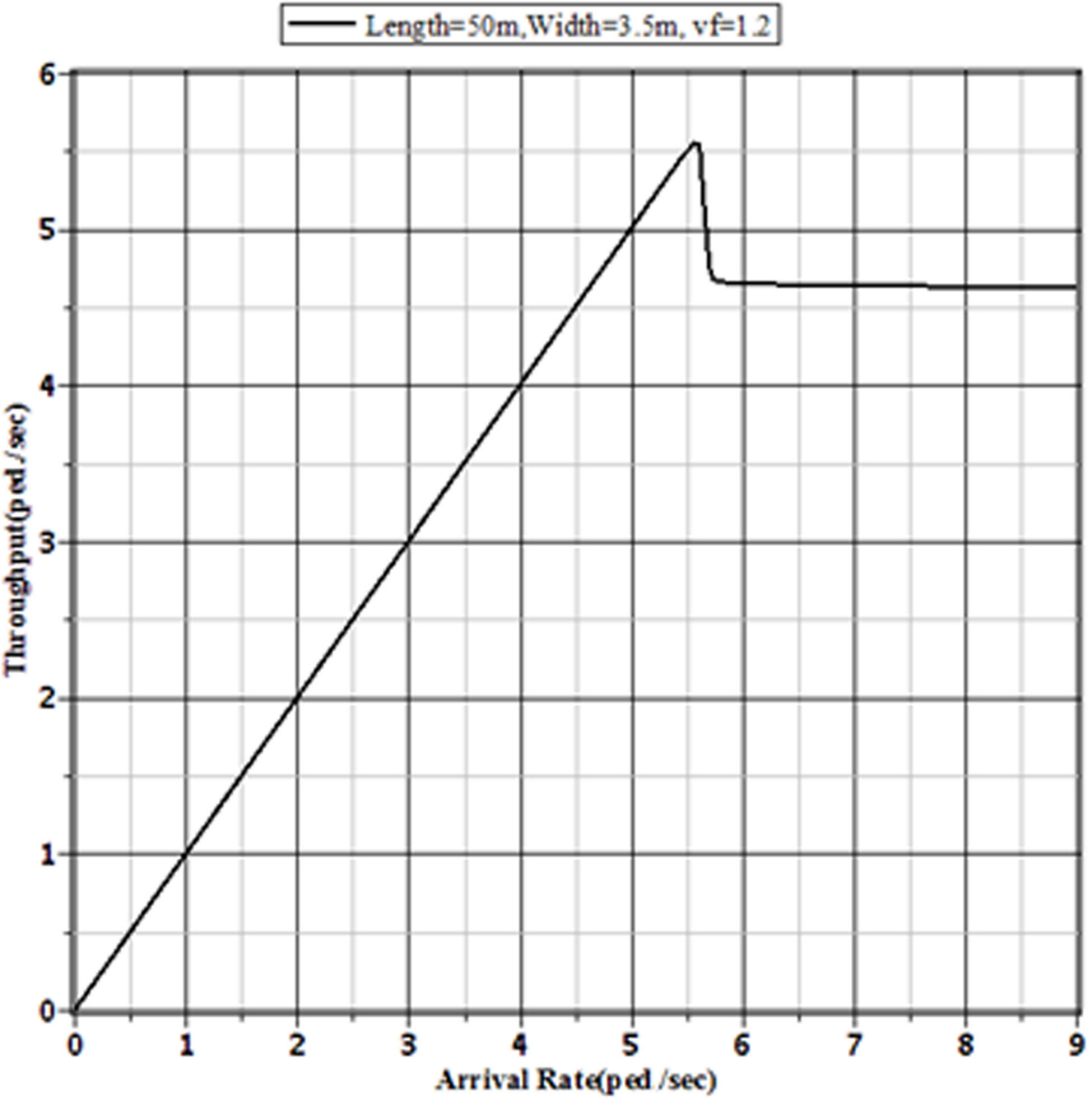
Throughputs of the Facility for Different Arrival Rates to a 50 m × 3.5 m Sidewalk with v_*f*_ = 1.2 m/sec.

**Fig 8:**
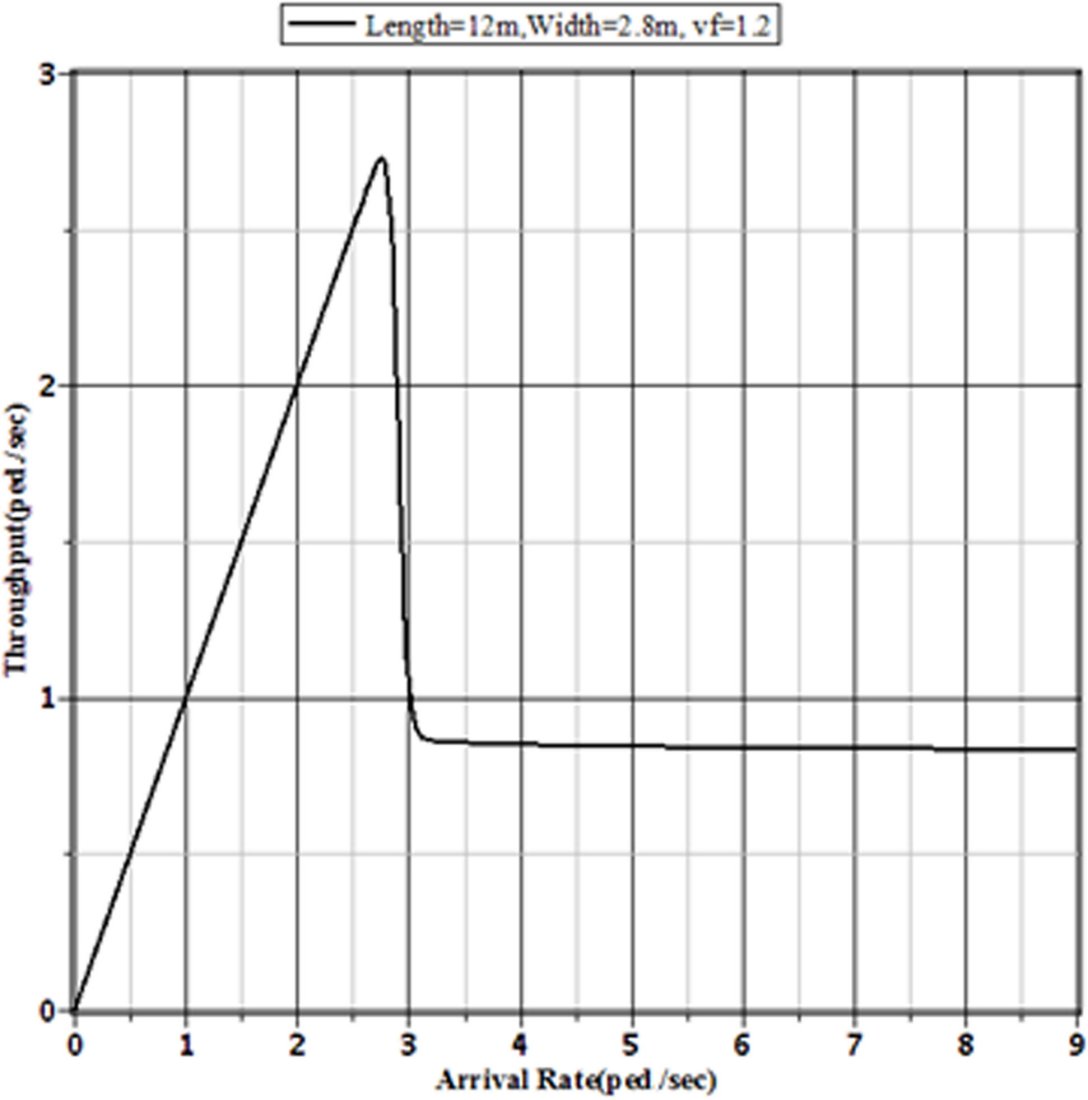
Throughputs of the Facility for Different Arrival Rates to a 12 m × 2.8 m Sidewalk with v_*f*_ = 1.2 m/sec.

**Fig 9:**
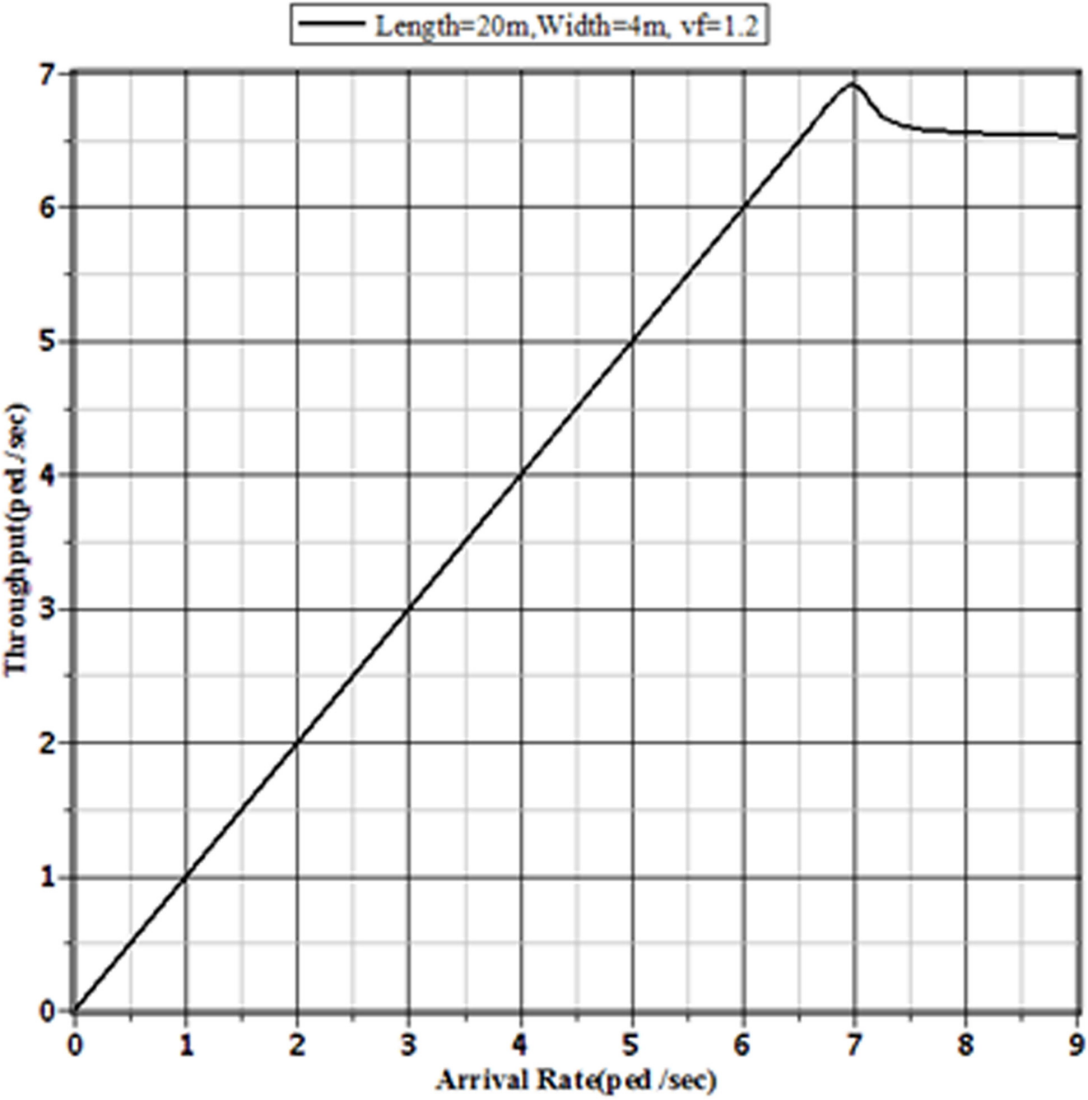
Throughputs of the Facility for Different Arrival Rates to a 20 m × 4 m Sidewalk with v_*f*_ = 1.2 m/sec.

Figs 7–9 show the effect of different pedestrian arrival rates on the pedestrian throughput in different sized sidewalks in Dhaka. From these figures, it is clear that the pattern of relationship between actual arrival rates and the corresponding throughputs are the same. Each of different sized sidewalks shows that the throughput approaches to a limit value with the increment of pedestrian arrival rate.

### 4.2 The Effect of Sidewalk Width on Performances

**Fig 10:**
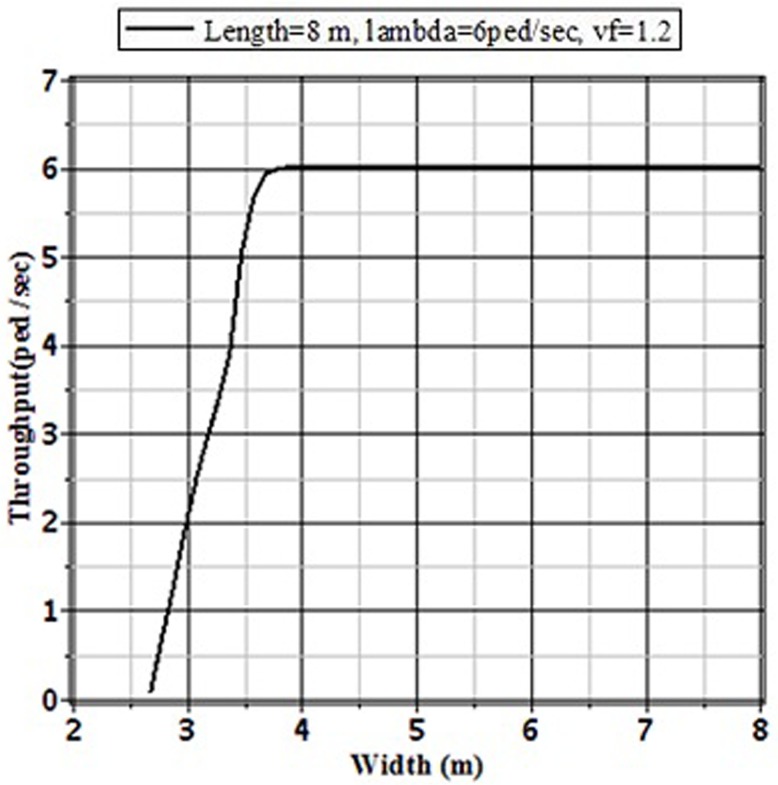
Throughputs of an 8 m Length Sidewalk for Different Sizes of Width with Arrival Rate *λ* = 6 ped./sec and *v*_*f*_ = 1.2 m/sec.

To study the influence of sidewalk width on the performances we can fix the values of pedestrian arrival rate to the facility, length of the facility, and the pedestrian free flow speed capacity. Then we can vary the value of width of the facility to identify the most advantageous width size of the facility that will optimize the pedestrian movement performance measures on the facility. In Table 2 different performances of a sidewalk of 8 m length at which pedestrians in Dhaka are arrived at a rate 6 ped./sec are presented by varying the width size. In addition, Fig 10 shows the pedestrian throughputs as a function of the width size of the facility. For the width size that is less than or equal to 2.67 m, there are no valid values for the performances exist. This is for the reduction of 1.07 m from the practical width to calculate the effective width of the facility and for the requirement of minimum 1.60 m width for passing two pedestrians in a bidirectional flow. Thus, the minimum width of a sidewalk should be greater than 2.67 m. From the Table 2 and Fig 10, it is clear that performances are optimized at the width size of around 3.8 m. At this width the effective arrival rate or throughput equal to actual arrival rate, the balking probability and the number of pedestrians in the queue start to disappear, and the average time that that a pedestrian needs to pass the facility is approximately equal to the result of the length (8 m) divided by pedestrian free flow speed capacity (1.20 m/sec). Thus, for the selected sidewalk at which pedestrians in Dhaka are arrived at a rate 6 ped./sec, the increment of width beyond 3.8 m is not justified the against monetary cost and the use of valuable land.

**Table 2:** Performances of an 8 m length sidewalk for different sizes of width with arrival rate *λ* = 6 ped./sec and *v*_*f*_ = 1.2 m/sec.

Width	Balking Probability	Pedestrians in Queue	Average time for a pedestrian	Throughput
(*W*)	(*p*_*Balk*_)	*E*(*Q*)	*E*(*T*)	(*θ*)
2.68	0.98913	33.98871	1042.47730	0.06522
3	0.64517	37.42101	35.42542	2.12901
3.5	0.14027	31.18448	14.45239	5.15838
3.6	0.04269	16.18059	10.21903	5.74384
3.7	0.00725	6.08259	7.97120	5.95651
3.8	0.00030	1.49344	7.03172	5.99817
3.9	0.00004	0.79696	6.87580	5.99977
4	0.00000	0.46719	6.79735	5.99997
4.1	0.00000	0.28660	6.75163	6.00000
4.5	0.00000	0.02905	6.68032	6.00000
5	0.00000	0.00150	6.66834	6.00000

### 4.3 The Effect of Sidewalk Length on Performances

To examine the influence of sidewalk length on the performances we can vary the length of the sidewalk for the given values for pedestrian arrival rate to the facility, width of the facility, and the pedestrian free flow speed capacity. In Tables 3 and 4, by varying the length size, different performances of the sidewalks of 3.5 m length and 4 m length at which pedestrians in Dhaka are arrived at the rates 8 ped./sec and 10 ped./sec, respectively, are presented. From the tables, it is observed that although both the number of pedestrians in the queue and the average time that a pedestrian needs to pass the facility increase with the increment of length, the changes in the balking probability and the throughput are not significant. This is because while the increment of length increases the capacity of the facility, the benefit of it is detrimentally affected by the number of pedestrians in the queue and the increase of travel time for a pedestrian to pass the facility. Thus, the effect of sidewalk length on the probabilities in Eqs 8 and 9 and on the main performances (balking probability and throughput) is cancelled out. Hence, the length of a sidewalk should be determined based on the function and purpose of it; whereas the size of width should be determined to optimize the performances of pedestrian movements.

**Table 3:** Performances of a 3.5 m width sidewalk for different sizes of length with arrival rate *λ* = 8 ped./sec and *v*_*f*_ = 1.2 m/sec.

Length	Balking Probability	Pedestrians in Queue	Average time for a pedestrian	Throughput
(*L*)	(*p*_*Balk*_)	*E*(*Q*)	*E*(*T*)	(*θ*)
5	0.38588	26.24613	11.04144	4.91293
10	0.40756	53.48653	22.88989	4.73950
15	0.41435	80.55011	34.69410	4.68524
20	0.41767	107.57957	46.48954	4.65867
25	0.41964	134.59658	58.28177	4.64290
30	0.42094	161.60765	70.07246	4.63246
35	0.42187	188.61544	81.86230	4.62503
40	0.42257	215.62121	93.65162	4.61947
45	0.42073	243.61209	105.43688	4.63417
50	0.42140	270.61705	117.22601	4.62881
75	0.42340	405.63171	176.16898	4.61280

**Table 4:** Performances of a 4 m width sidewalk for different sizes of length with arrival rate *λ* = 10 ped./sec and *v*_*f*_ = 1.2 m/sec.

Length	Balking Probability	Pedestrians in Queue	Average time for a pedestrian	Throughput
(*L*)	(*p*_*Balk*_)	*E*(*Q*)	*E*(*T*)	(*θ*)
5	0.34144	28.99111	9.10943	6.58560
10	0.34598	60.07030	18.66450	6.54024
15	0.34751	91.09648	28.21461	6.52486
20	0.34829	122.10946	37.76338	6.51715
25	0.34875	153.11720	47.31163	6.51251
30	0.34909	184.12235	56.85959	6.50941
35	0.34928	215.12601	66.40741	6.50720
40	0.34945	246.12876	75.95512	6.50554
45	0.34958	277.13089	85.50277	6.50424
50	0.34968	308.13259	95.05038	6.50321
75	0.34999	463.13769	142.78804	6.50011

### 4.4 The Effect of Pedestrian Personal Capacity on Traffic Congestion

**Fig 11:**
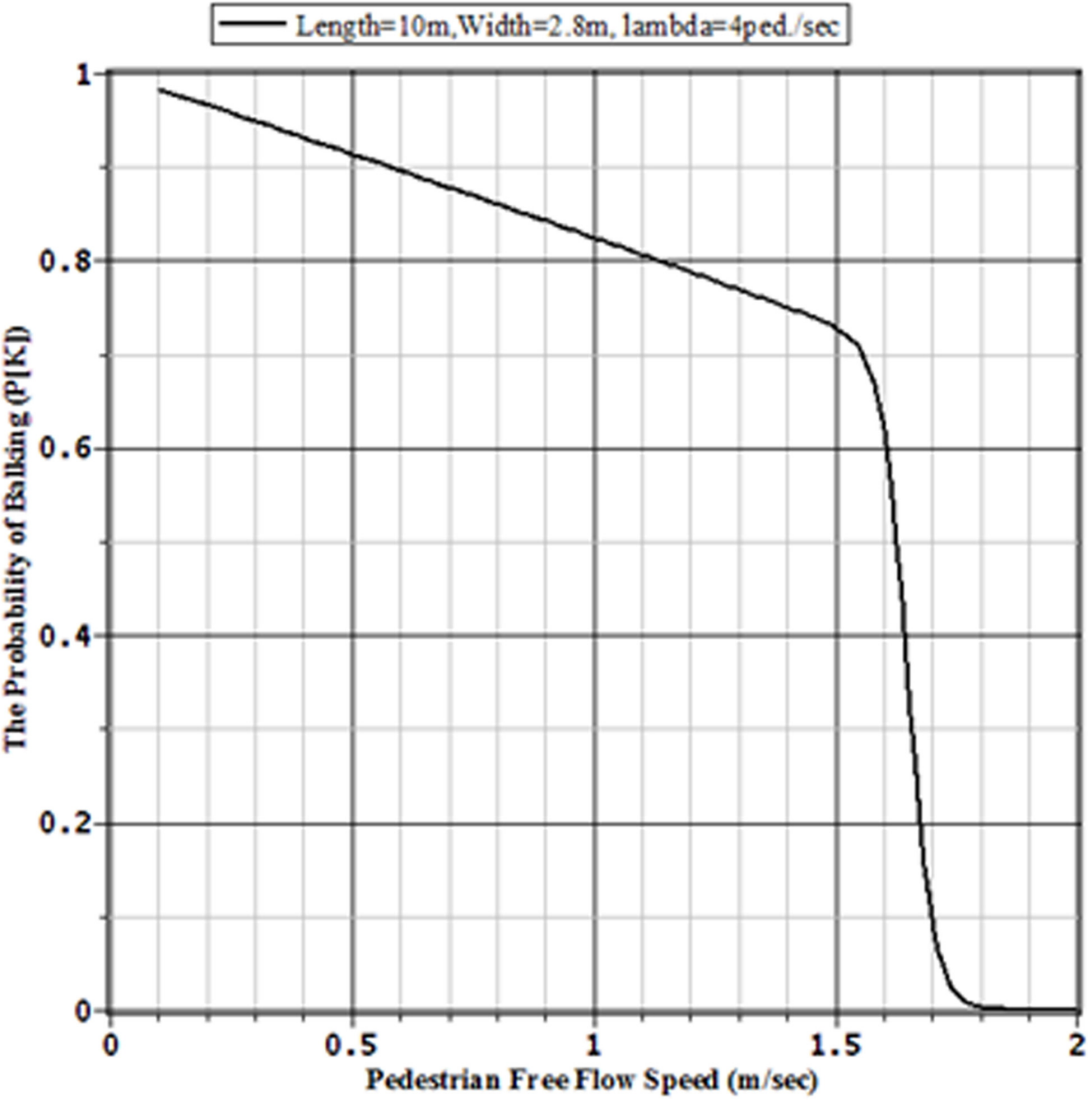
Balking Probabilities of a 10 m × 2.8 Sidewalk for Various Pedestrian Free Flow Capacities with Arrival Rate *λ* = 4 ped./sec.

**Fig 12:**
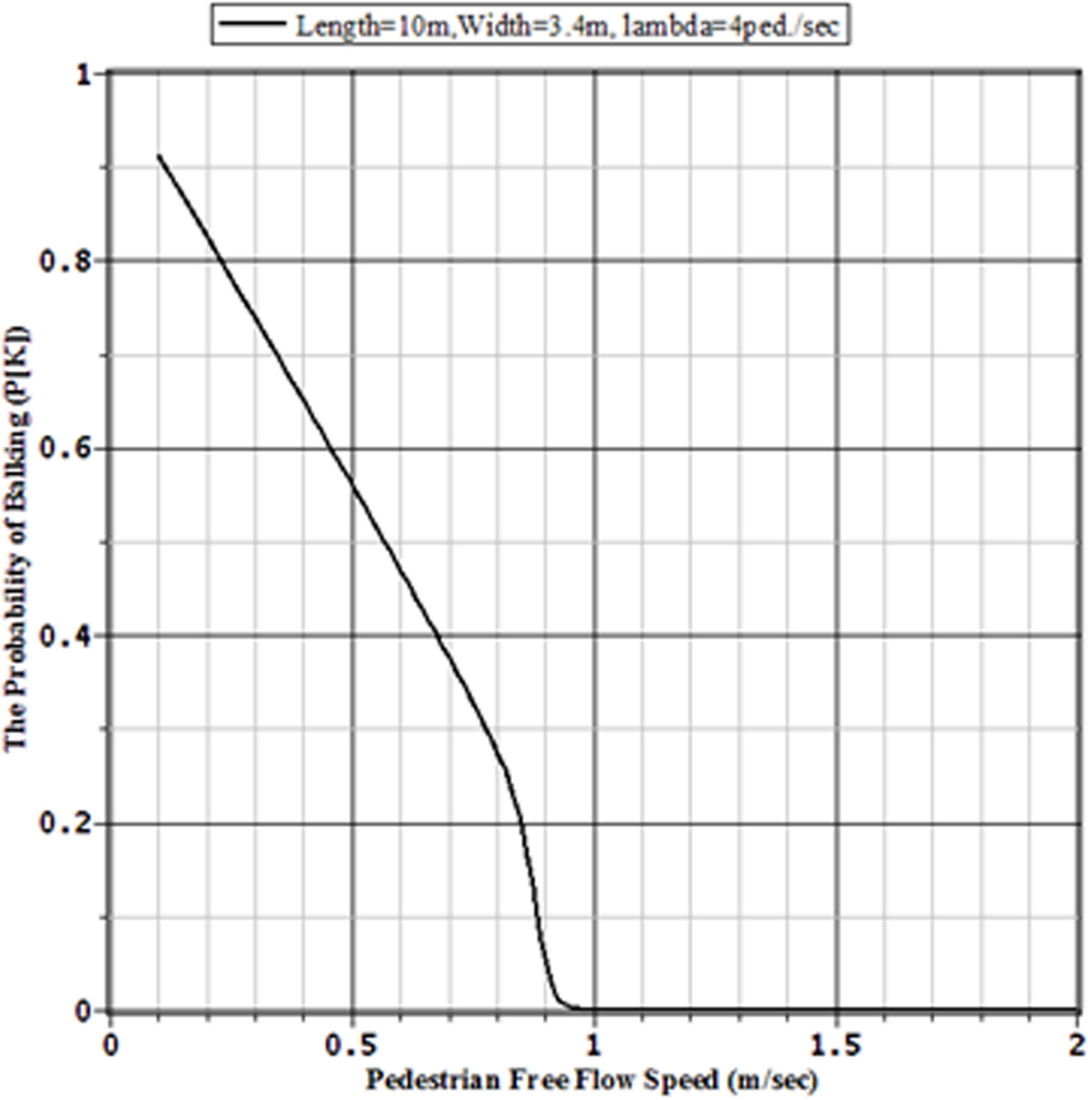
Balking Probabilities of a 10 m × 3.4 m Sidewalk for Various Pedestrian Free Flow Capacities with Arrival Rate *λ* = 4 ped./sec.

Finally, to analyse the effect of pedestrian personal capacity on the traffic congestion and the sizes of facilities we can vary the pedestrian free flow speed capacity for the given values for the pedestrian arrival rate to the facility, the length and width of the facility. Tables 5 and 6 together with Figs 11 and 12, respectively, show how the values of probability of balking of two sidewalks of 10 m by 3 m and 10 m by 5 m, in both of which pedestrians are arrived at the rates 4 ped./sec, are decreased with the increment of pedestrian personal capacity to walk. From the tables and figures it is obvious that, for congestion free pedestrian movements, pedestrians with lower capacity require wider width of sidewalk than that is needed for higher capable pedestrians. This implies that, for faster and congestion free pedestrian movements, the sidewalks in Bangladesh should be wider than those of width required for the sidewalks in Western countries. This is for the reason that Bangladeshi pedestrians are slower than those of Western countries [3]. This finding supports the findings of Rahman et al. [3,18] that the Western design codes for the pedestrian facilities should not be directed used in the countries like Bangladesh. The walking infrastructures should be constructed based on the local pedestrian characteristics.

**Table 5:** Performances of a 10m × 2.8 m sidewalk for various pedestrian free flow capacities with arrival rate *λ* = 4 ped./sec.

Pedestrian free flow	Balking Probability	Pedestrians in Queue	Average time for a pedestrian	Throughput
(*v*_*f*_)	(*p*_*Balk*_)	*E*(*Q*)	*E*(*T*)	(*θ*)
0.50	0.91350	87.90398	254.05892	0.34600
1.45	0.73463	87.59600	84.10669	1.04149
1.50	0.72795	87.41575	80.33184	1.08818
1.55	0.70511	86.31087	73.17135	1.17957
1.60	0.62011	79.86029	52.55499	1.51956
1.70	0.09836	34.09914	9.45471	3.60658
1.75	0.01788	26.04517	6.62984	3.92848
1.80	0.00301	23.89344	5.99141	3.98795
1.85	0.00051	22.92971	5.73537	3.99795
1.90	0.00009	22.20918	5.55279	3.99964
2	0.00000	20.96484	5.24122	3.99999

**Table 6:** Performances of a 10m × 3.4 m sidewalk for various pedestrian free flow capacities with arrival rate *λ* = 4 ped./sec.

Pedestrian free flow	Balking Probability	Pedestrians in Queue	Average time for a pedestrian	Throughput
(*v*_*f*_)	(*p*_*Balk*_)	*E*(*Q*)	*E*(*T*)	(*θ*)
0.50	0.56104	105.20228	59.91618	1.75582
0.75	0.32028	103.72747	38.66301	2.68286
0.80	0.27637	102.63919	35.46001	2.89451
0.85	0.19928	96.46151	30.11708	3.20288
0.90	0.05368	65.10045	17.11830	3.78528
0.95	0.00308	45.93320	11.51874	3.98769
1.00	0.00012	41.61121	10.40409	3.99950
1.10	0.00000	37.22281	9.30570	3.99999
1.50	0.00000	26.91923	6.72981	4
2	0.00000	20.08781	5.02195	4

## Conclusions

Optimal pedestrian flow control and the proper design and sizing of facilities are challenging tasks to traffic operators and planners. In this study, we have utilized the realistic relationships among the pedestrian traffic flow variables to formulate a credible analytical model based on M/M/c/K state-dependent queuing system. The model incorporates the most important real factors that have significant influences on the operating characteristics and the performances of pedestrian flows on sidewalk facilities. With the model we have found that pedestrian movements with different arrival rates on different seized walking facilities have some analogous characteristics. For the fixed values for length, width of the facility, and the pedestrian free flow speed capacity, the traffic operators can identify an optimal pedestrian arrival rate at which the throughput is maximized and there is no overflow on the facility. The analysis based on the formulated model also point out that the length of a sidewalk should be determined based on the function and purpose of it. However, the size of width should be determined to optimize the performances and smooth movement of pedestrians. Finally, an analysis of the pedestrian free flow personal capacity does not validate the sustainability of adaptation of Western design codes for the pedestrian facilities in the countries like Bangladesh.

### Appendix

Kendall (1953) designed a convenient notation for denoting a queuing system, which has been generally accepted and is now rather standard throughout the queuing literature[23]. The notation contains a five-part descriptor *A/B/X/Y/Z*, where the first and the second symbols indicate in some way the interarrival and service time distributions, respectively. The third, the fourth and the fifth symbols indicate the number of servers, customers holding capacity limit of the system and the queue discipline, respectively. Table 7 presents some standard symbols for these characteristics. For example, the notation *D/M/3/∞/LCFS* indicate a queuing system with deterministic interarrival times, exponential service times, three parallel servers, no restriction on the maximum number of customers allowed in the system, and last- come, first- served queue discipline.

**Table 7:** Queuing Notation *A/B/X/Y/Z*.

Characteristic	Symbol	Explanation
	*M*	Exponential
	*D*	Deterministic
Interarrival-time distribution (*A*)	*E*_*k*_	Erlang type *k* (*k* = 1,2,…., ∞)
Service-time distribution (*B*)	*H*_*k*_	Mixture of *k* exponentials
	*PH*	Phase type
	*G*	General
Number of parallel servers (*X*)	1,2,…., ∞	
Restriction on system capacity (Y)	1,2,…., ∞	
	FCFS	First come, first served
	LCFS	Last come, first served
Queue discipline (*Z*)	RSS	Random selection for service
	PR	Priority
	GD	General discipline

In many situations the last two symbols are omitted. If the fourth symbol is not mentioned, it means that there is no limit in the capacity of the system (*Y* = ∞) and if the fifth symbol is not mentioned, it means that first-come, first-served (*Z = FCFS*) discipline is followed in the system. Thus *D/M/3* would be a queuing system with deterministic input, exponential service, three servers, no limit on system capacity, and first- come, first- served discipline.

## Supporting Information

S1 File Conference paper. (DOCX)

S2 File Codes. (DOCX)

## References

[1] Seneviratne P, Morrall J (1985) Level of service on pedestrian facilities. Transportation quarterly 39: 109–123.

[2] Braess D (1968) Uber ein Paradoxon aus der Verkehrsplanung. Unternehmensforschung, 12: 258–268, 1968. English translation in Braess.

[3] Rahman K, Ghani NA, Kamil AA, Mustafa A (2012) Analysis of pedestrian free flow walking speed in a least developing country: a factorial design study. Research Journal of Applied Sciences, Engineering and Technology 4: 4299–4304.

[4] Rahman K, Ghani NA, Kamil AA, Mustafa A (2013) Weighted regression method for the study of pedestrian flow characteristics in Dhaka, Bangladesh. Modern Applied Science 7: 17–30.

[5] Hu L, Jiang Y, Zhu J, Chen Y (2015) A PH/PH (n)/C/C state-dependent queuing model for metro station corridor width design. European Journal of Operational Research 240: 109–126.

[6] Yuhaski SJ, Smith JMG (1989) Modeling circulation systems in buildings using state dependent queueing models. Queueing Systems 4: 319–338.

[7] Mitchell DH, MacGregor Smith J (2001) Topological network design of pedestrian networks. Transportation Research Part B: Methodological 35: 107–135.

[8] Liu S, Lo S, Ma J, Wang W (2014) An agent-based microscopic pedestrian flow simulation model for pedestrian traffic problems. IEEE Transactions on Intelligent Transportation Systems 15.

[9] Ding N, Chen T, Zhang H, Luh PB (2015) Stair Evacuation Simulation Based on Cellular Automata Model Considering Social Forces Traffic and Granular Flow' 13: Springer pp. 145–153.

[10] Hao Q-y, Jiang R, Hu M-B, Wu Q-S. Unidirectional pedestrian flow in a lattice gas model coupled with game theory; 2011 IEEE pp. 1054–1058.

[11] van Woensel T, Cruz FR (2014) Optimal Routing in General Finite Multi-Server Queueing Networks. PloS one 9: e102075 doi: 10.1371/journal.pone.0102075 25010660 10.1371/journal.pone.0102075 PMC4092093

[12] Haight FA (1957) Queueing with balking. Biometrika 44: 360–369.

[13] Kumar BK, Parthasarathy P, Sharafali M (1993) Transient solution of anM/M/1 queue with balking. Queueing Systems 13: 441–448.

[14] Arita C (2009) Queueing process with excluded-volume effect. Physical Review E 80: 051119. 10.1103/PhysRevE.80.051119 20364959

[15] Bojkovic Z, Bakmaz M, Bakmaz B (2010) Originator of teletraffic theory. Proceedings of the IEEE 98: 123–127.

[16] Chung S-P, Kashper A, Ross KW (1993) Computing approximate blocking probabilities for large loss networks with state-dependent routing. IEEE/ACM Transactions on Networking (TON) 1: 105–115.

[17] Polus A, Schofer JL, Ushpiz A (1983) Pedestrian flow and level of service. Journal of transportation engineering 109: 46–56.

[18] Rahman K, Ghani NA, Kamil AA, Mustafa A (2013) Modelling pedestrian travel time and the design of facilities: a queuing approach Accepted for Publication. 10.1371/journal.pone.0063503 PMC3655143 23691055

[19] Older S (1968) Movement of pedestrians on footways in shopping streets. Traffic Engineering & Control 10: 160–163.

[20] Council NR (2000) Highway capacity manual Transportation Research Board, Washington, DC.

[21] Al-Azzawi M, Raeside R (2007) Modeling pedestrian walking speeds on sidewalks. Journal of Urban Planning and Development 133: 211–219.

[22] Navin F, Wheeler R (1969) Pedestrian flow characteristics. Traffic Engineering, Inst Traffic Engr 39: 30–36.

[23] Gross D (2008) Fundamentals of queueing theory: John Wiley & Sons.

