# Supplementary material for: An M/M/c/K State-Dependent Model for Pedestrian Flow Control and Design of Facilities
Source: PLoS One. 2015 Jul 21;10(7):e0133229. doi: 10.1371/journal.pone.0133229 (PMC4511190; doi:10.1371/journal.pone.0133229)
Supplement: S1 File — (DOCX) [file pone.0133229.s001.docx]

**Development of an M/M/c/K State-Dependent Model for the Management of Pedestrian Movements on Sidewalks**

**Khalidur Rahman^1,3*^, Noraida Abdul Ghani^1^, Anton Abdulbasah Kamil^1^,**

**Adli Mustafa^2^**

^1^Mathematics Section, School of Distance Education, Universiti Sains Malaysia 11800 USM, Penang, Malaysia

^2^School of Mathematical Sciences, Universiti Sains Malaysia 11800 USM, Penang, Malaysia

^3^Department of Statistics, School of Physical Sciences, Shahjalal University of Science and Technology, Sylhet-3114, Bangladesh

**ABSTRACT:** On sidewalks in the Central Business Districts (CBD) of a city, overflow of pedestrians is the main source of queuing delay which is caused by the capacity of sidewalks and management practices. The concept of flow management can be used to avoid the building of extreme queues of pedestrians on sidewalks in the CBD. Thus, in this paper, the application of M/M/c/K state-dependant queuing models for managing the flow of pedestrians and the design of sidewalks in the CBD is discussed. The paper also derives some performance measures that are useful for such management and the design of sidewalks in the CBD.

**Keywords:** Pedestrians; Sidewalks; Management; Design; State-dependent Model.

*Corresponding author: [khalid_sust@yahoo.com](mailto:khalid_sust@yahoo.com)

**1. Introduction**

Flow management is one of the common means to ensure proper quality of service with limited facilities in transportation and communication industry. An efficient flow management approach is the one that adjusts the input flow based on the appropriate operating characteristics to provide optimum performances. Therefore, the real factors that have significant influences on the operating characteristics and the performances should be incorporated as much as possible in the flow management approach. The incorporation and the assessment of influences are usually done by using an analytical model. However, the opportunity to find an appropriate analytical tool is very limited as few real-world situations conform to the requisite assumptions.

The Central Business District (CBD) of a city is ideally suited for the provision of efficient pedestrian networks. Thus, the pedestrian facilities in CBD areas should not turn into bottlenecks/ high congestions. On the other hand, although the increment of capacity (additional line) eases the pedestrian jam, the necessity of additional line on the valuable land in CBD should be justified against the monetary cost and based on how the users rank delays due to congestion (Seneviratne & Morrall, 1985). Braess (1968) noticed that, in the congested traffic condition, addition of line to the capacity leads to an increase in the average travel time for everyone. This is because self-serving individuals cannot refrain themselves from utilizing the additional capacity, even though such utilization leads to deterioration in the average travel time. Thus, one way to avoid the bottlenecks is to manage the flow based on the local pedestrian flow characteristics (Rahman, Ghani, Kamil, & Mustafa, 2012, 2013a) and to design the pedestrian facilities according to their demand for flow. The pedestrian flows and facilities should also be analysed with an appropriate analytical tool that can incorporate important determinants and functional factors. However, there are a small number of researches that have devoted to manage the pedestrian flow as well as the capacity based design of pedestrian facilities. Among those, Yuhaski & Smith (1989) and Mitchell & MacGregor Smith (2001) presented comprehensive analyses of pedestrian facilities and developed an analytical approximation methodology based on M/G/C/C state dependent queuing models to compute certain performance measures for pedestrian facilities. However, to overcome some limitations and to incorporate some important determinants and functional factors, as mentioned in the next section and thereafter, in this study we will develop the M/M/c/K state-dependent queuing models to manage pedestrian flows and capacity based design of pedestrian facilities in open outdoor walking environment e.g. sidewalks.

The rest of the paper is organized in the following manner. The reasons for using M/M/c/K state dependent queuing models and the pedestrian speed model that can represent the state dependent services on sidewalks are presented in Section 2. The background to the use and development of the M/M/c/K state-dependent pedestrian flow management model to analyze a single link of a network of sidewalks is provided in Section 3, which could be extended to analyse pedestrian flows through the network. The paper ends with conclusion and further research.

**2. Queuing and Congestion Models for Sidewalks**

Crowd on the sidewalks in a CBD sometimes brings the pedestrian movements to a standstill. In such situations, a pedestrian could not freshly join the queue on the facility because the facility is already at the capacity (i.e. balk). Balking is not only a common phenomenon in the pedestrian movements, but it is also frequently observed in vehicle traffic, machine repair models etc. In 1917 Danish mathematician A K Erlang gave a formula for loss and waiting time based on M/M/s/s system, which was soon used by many telephone companies in different countries (Bojkovic, Bakmaz, & Bakmaz, 2010; Chung, Kashper, & Ross, 1993). The loss formula is known as Erlang’s B formula, where B stands for blocking, and can be used for estimating the probability of balking in telephone, cable etc. This loss formula has also been used by Yuhaski & Smith (1989) and Mitchell & MacGregor Smith (2001) in the performance measures and the planning of pedestrian facilities and networks. However, the following reasons have stimulated to adopt the M/M/c/K system based queuing models rather than M/G/C/C queuing models for the design and analysis of pedestrian networks in open outdoor walking facilities:

1. The M/G/C/C state dependent queuing models consider that the queue consists entirely of the walking facility without any buffer space. Such consideration may be applicable in an emergency evacuation from a building or in circuit switching. However, it is not reasonable for the uninterrupted and moving pedestrians in an open outdoor walking facility/ sidewalk.
2. The waiting/lingering time, and the lateral spacing required for the movements of a pedestrian on a walking facility are not explicitly reflected in the formulation of the corresponding congestion models.
3. The congestion models for M/G/C/C state-dependent queuing models do not support the observation of Polus, Schofer, & Ushpiz (1983), that is to say, up to the densities of about 0.6 ped./m^2^ the free flow condition remains valid on sidewalk facilities.

The M/M/s system based congestion models as derived in Eq. (1) based on the model developed by Rahman et al. (2013b), which has been empirically validated, and M/M/c/K state-dependent pedestrian flow models, as formulated in Section 3, could triumph over these shortcomings to study the pedestrian movements and flow management on sidewalk facilities, and designing of such facilities.

*2.1 Pedestrian Walking Speeds on Sidewalks*

Many studies on pedestrian movements have been carried out under different conditions. The studies found that the patterns of pedestrian movements under the normal and emergency conditions are not same. However, there are some common personal factors such as age, gender, intelligence, and physical fitness of a pedestrian have significant influence on the pedestrian speeds in any walking condition and environment. Since pedestrian free flow speed is mainly influenced by the pedestrian variables or personal attributes, the inclusion of most common factors to the modelling of pedestrian movements can be done by bringing free flow speed to the corresponding congestion models.

In a public walkway facility, the usual movements of a pedestrian are hindered by the presence of other pedestrians (Older, 1968). Thus, on the sidewalks in a CBD, interaction with other pedestrians is the most important factor that influences the pedestrian speed and flow. People moving on the sidewalks are not always in huge numbers, but certainly at high densities. On the sidewalks, pedestrians usually travel at a maximum density of 1.55 ped./m^2^ (normal capacity), whereas pedestrian free flow speeds start to decline at a density of 0.6 ped./m^2^ and ‘usual jam’ (the facility is at the capacity) occurs at densities of about 3.32 ped./m^2^ (Polus et al., 1983; Rahman et al., 2013a, 2013b). As far as continuous pedestrian movements on sidewalks is concerned, ‘usual jam’ leading to ‘solid jamming’ seemingly takes place at densities in the range 4 to 5 ped./m^2^, which is very rare to be occurred. Rahman et al. (2013b) have developed a non-linear analytical model for pedestrian speeds on sidewalks, which supports the above mentioned empirical results and can be expressed as Eq. (1) as a function of the number of pedestrians on a sidewalk.

*2.2 Congestion Model for Sidewalks*

In a congested situation, the three variables that completely describe the pedestrian traffic flows are speed, flow and density. Pedestrian flows moving on a facility represent pedestrians’ demand. Depending on the volume of flow and other factors (e.g. personal attributes) speed and density will fluctuate. Confronting the demand for flow and the capacity of the facility (supply) determines the operating characteristics and the performances of a pedestrian infrastructure under investigation. Speed is a key measure to the quality of service (service rate) provided to the pedestrians on the facility and as such determines the effectiveness of the facility infrastructure (Council, 2000). In addition to personal attributes, the average speed of pedestrians is influenced by many other factors including the purpose of the journey, the physical nature of the walkway, the nature of the surrounding area, and weather (Al-Azzawi & Raeside, 2007). However, for the purpose of capacity analysis only concentration (the number of pedestrians in the facility) should be considered (Navin and Wheeler, 1969). Hence, the speed-density relationship on sidewalks developed by Rahman et al. (2013b) has been adopted in this study and it can be expressed as the following as a function of the number of pedestrians on a sidewalk.

(1)

| where | *v_m_* | = | average walking speed of *m* pedestrians on the facility (m/sec); |
| --- | --- | --- | --- |
|  | *v_f_* | = | average free flow speed of a pedestrian (m/sec); |
|  | *c* | = | *1.55*W*L* = the normal capacity of a sidewalk facility (ped.); |
|  | *W* | = | the width of the facility (m); |
|  | *L* | = | the length of the facility (m); |
|  | *m* | = | number of pedestrians on the facility, *m* = 1, 2, …,*c*,…,K(= 2*c*); |
|  | *s* | = | $\frac{W-1.07}{b}$= number of pedestrian lanes on the facility, where 1.07 m of width is reduced to calculate the effective width of the facility (Navin and Wheeler, 1969) and |
|  | *b* | = | lateral spacing required for a pedestrian to move on the facility (m) = 0.8m (Council, 2000). |

As mentioned in sub-section 2.1, pedestrians on sidewalks usually move at maximum density of 1.55 ped./m^2^. Therefore, the normal capacity, *c*, is equal to 1.55 times the area of the facility in square meters (m^2^). Hence, in the above, *c* is expressed as 1.55*W*L*.* In addition, the ‘usual jam’ (the facility is at the capacity) occurs at densities of about 3.32 ped./m^2^. Thus, it is reasonable to consider that the facility will be at jam capacity when there are more 2c pedestrians on the facility. Therefore, in the above, it is considered that the highest number of pedestrians on the facility could be up to K = 2*c*. For illustration and experimentation purposes, we will be confined ourselves to the above considerations and assumptions.

**3. Analytical Model for a Single Sidewalk**

It is mentioned in the sub-section 2.2 that pedestrian speed, flow and density (the number of pedestrians on a facility) completely describe the pedestrian traffic flows in a congested situation. Thus, it is always favourable if the performance measures of a pedestrian facility could be formulated based on these three variables. In the study of stochastic nature of pedestrian flows, when two of the three variables are known, the traffic operator can manage the third one to meet performance measures to the targeted magnitudes. Based on the capacity of a facility, the pedestrian flow ascertains the operating characteristics and the performances of an infrastructure under investigation. Thus, the managing of pedestrian flows for given speeds and the number of pedestrians on a facility is more convenient in terms of sharply response to the stochastic changes in pedestrian traffic conditions.

In the development of analytical models for understanding the stochastic nature of pedestrian flows, we can define a single sidewalk as a station where pedestrians are served. It is considered that the sidewalk has *c* servers in the normal capacity (as discussed in the previous section), which provide facilities to the pedestrians to pass the sidewalk without overflowed. A pedestrian can be both an output and an input to the queuing system within the sidewalk. Here, we assume that pedestrians enter the sidewalk in accordance with a Poisson process with rate $\lambda$(and thus the inter-entrance times are exponentially distributed), enter the sidewalk if it is not in jam capacity K, and then spend an exponential amount of time on the sidewalk with rate $\mu_{m}$ being served. The service time is equal to the travel time required for a pedestrian to pass the entire length of the sidewalk. The travel time and hence the service rate,$\mu_{m}$, is state dependent as the travel time of each pedestrian within the sidewalk depends on the number of prevailing pedestrians on the sidewalk. It is assumed that, at each moment of time, pedestrians are uniformly distributed over the sidewalk. Thus, for *m* pedestrians on the sidewalk, the service rate will be a function of *m* i.e.$f(m)$. Since the sidewalk normal capacity is *c* and jam capacity is K, we can model the stochastic nature of pedestrian flows on a sidewalk with a queuing model. Thus, our considered model, in Kendall notation, could be described as M/M/c/K.

From the balance equations for M/M/c/K queuing system, we have the steady-state probabilities *p_m_* (m = 1, 2…,c,…..K) of *m* pedestrians on the sidewalk as

$p_{m}=\left\{\begin{matrix} \frac{\lambda_{0}\lambda_{1}............\lambda_{m-1}}{\mu_{1}\mu_{2}...........\mu_{m}}p_{0} for 1\leq m\leq c \\ \\ \frac{\lambda_{0}\lambda_{1}............\lambda_{m-1}}{\mu_{1}\mu_{2}.....\mu_{c}(\mu_{c})^{m-c}}p_{0} for c\leq m\leq K \end{matrix}\right.$ (2)

From the normalizing condition for *p_0_*, probability of no pedestrian on the sidewalk, we have

$p_{0}=\left(1+\sum_{m=1}^{c-1} \frac{\lambda_{0}\lambda_{1}............\lambda_{m-1}}{\mu_{1}\mu_{2}...........\mu_{m}}+\sum_{m=c}^{K} \frac{\lambda_{0}\lambda_{1}............\lambda_{m-1}}{\mu_{1}\mu_{2}.....\mu_{c}(\mu_{c})^{m-c}}\right)^{-1}$ (3)

In the flow management modelling, we consider that the arrival rates will be controlled (for example, by using a roundabout on the middle or in the entry and exit points of the sidewalk) and hence arrival rates are not influenced by the number of prevailing pedestrians, *m*, on the sidewalk. We, therefore, assume that the arrival rates of flows are constant such that $\lambda=\lambda_{0}=\lambda_{1}=.......=\lambda_{c}=.....=\lambda_{K-1}$ and then we have from Eqs. (2) and (3)

(4)

and

(5)

where $\mu_{i}$, for i = 1, 2…..*m*, is a function of i, the number of pedestrians on the sidewalk. Since the number of prevailing pedestrians affects the average pedestrian speed/travel time and hence the service rates, we can use the non-linear congestion model of Eq. (1) to describe $\mu_{m}$ on a single sidewalk. Note that the service rate, *r_m_*, when there are *m* pedestrians on the sidewalk, is equal to the inverse of the average time that is required for a pedestrian to traverse the length of the sidewalk; therefore,

$r_{m}=\frac{v_{m}}{L}$ (6)

Since, in our consideration, *m* servers simultaneously serve on the sidewalk facility, we will have the overall service rate for *m* pedestrians as

$\mu_{m}=mr_{m}=m\frac{v_{m}}{L}$ (7)

By substituting the expression for$\mu_{m}$, from Eq. (7) into Eqs. (4) and (5), we obtain the steady-state probabilities as

(8)

and

(9)

where $E\left(S\right)=\frac{L}{v_{f}}$is the expected service time/ traverse time for a pedestrian in free flow condition in a sidewalk of length L and

is the ratio of average speed of *m* pedestrians on the sidewalk to that of speed in free flow condition. Since the speed has been expressed as a function of number of pedestrians or density, for a particular number of pedestrians on a given facility, the steady-state probabilities and the corresponding performance measures (as will be discussed in the next section) will depend on the arrival rates i.e. on the pedestrian flows.

**4. Conclusion and Further Research**

The use of M/M/c/K queuing models for analysing a single sidewalk allows us to compute certain performance measures in equilibrium condition. The most relevant performance measures include

1. The probability of balking ($p_{Balk}$) is equal to *p_m_* where m equals K,
2. $E(Q)=\sum_{m=c+1}^{K} (m-c)p_{m}$is the average number of pedestrians waiting in the queue in the equilibrium condition,
3. $E(T)=\frac{\sum_{m=1}^{K} mp_{m}}{\theta}$ is the expected amount of time a pedestrian spends on the facility,
4. $\theta=\lambda(1-p_{Balk})$is the pedestrian effective arrival to the sidewalk or throughput through the sidewalk.

The most important factors that influencing the behaviour of pedestrian flows and the related performances of a walking facility include pedestrian arrival flow rate,$\lambda$, length of the facility L, width of the facility W and pedestrian personal capacity *v_f_*.

What is intended to do in a further study is to examine the effects of these factors on performances. Such examinations will be useful for proper management and the design of sidewalks in the CBD.

**Acknowledgement**

This study was supported by the LRGS Grant Scheme [203/PTS/6728002], Ministry of Higher Education, Malaysia and the Research University (RU) Grant Scheme, [Acct. No.: 1001/PJJAUH/811097], Universiti Sains Malaysia. Khalidur Rahman wishes to thank Universiti Sains Malaysia for the financial support (USM Fellowship).

**References**

Al-Azzawi, M., & Raeside, R. (2007). Modeling pedestrian walking speeds on sidewalks. Journal of Urban Planning and Development, 133(3), 211-219.

Bojkovic, Z., Bakmaz, M., & Bakmaz, B. (2010). Originator of teletraffic theory. Proceedings of the IEEE, 98(1), 123-127.

Braess, D. (1968). Uber ein Paradoxon aus der Verkehrsplanung. Unternehmensforschung, 12: 258–268, 1968. English translation in Braess.

Chung, S.-P., Kashper, A., & Ross, K. W. (1993). Computing approximate blocking probabilities for large loss networks with state-dependent routing. IEEE/ACM Transactions on Networking (TON), 1(1), 105-115.

Council, N. R. (2000). Highway capacity manual: Transportation Research Board, Washington, DC.

Mitchell, D. H., & MacGregor Smith, J. (2001). Topological network design of pedestrian networks. Transportation Research Part B: Methodological, 35(2), 107-135.

Navin, F., & Wheeler, R. (1969). Pedestrian flow characteristics. Traffic Engineering, Inst Traffic Engr, 39(June), 30-36.

Older, S. (1968). Movement of pedestrians on footways in shopping streets. Traffic Engineering & Control, 10(4), 160-163.

Polus, A., Schofer, J. L., & Ushpiz, A. (1983). Pedestrian flow and level of service. Journal of transportation engineering, 109(1), 46-56.

Rahman, K., Ghani, N. A., Kamil, A. A., & Mustafa, A. (2012). Analysis of pedestrian free flow walking speed in a least developing country: a factorial design study. Research Journal of Applied Sciences, Engineering and Technology, 4(21), 4299-4304.

Rahman, K., Ghani, N. A., Kamil, A. A., & Mustafa, A. (2013a). Modelling pedestrian travel time and the design of facilities: a queuing approach Accepted for Publication.

Rahman, K., Ghani, N. A., Kamil, A. A., & Mustafa, A. (2013b). Weighted regression method for the study of pedestrian flow characteristics in Dhaka, Bangladesh. Modern Applied Science, 7(4), 17-30.

Seneviratne, P., & Morrall, J. (1985). Level of service on pedestrian facilities. Transportation quarterly, 39(1), 109-123.

Yuhaski, S. J., & Smith, J. M. G. (1989). Modeling circulation systems in buildings using state dependent queueing models. Queueing Systems, 4(4), 319-338.
