# Supplementary material for: An M/M/c/K State-Dependent Model for Pedestrian Flow Control and Design of Facilities
Source: PLoS One. 2015 Jul 21;10(7):e0133229. doi: 10.1371/journal.pone.0133229 (PMC4511190; doi:10.1371/journal.pone.0133229)
Supplement: S2 File — (DOCX) [file pone.0133229.s002.docx]

**The Required Codes for Figures and Tables**

># Following codes were used in Maple to analyze the effect of pedestrian arrival rate on an 8 m by 3 m sidewalk in Dhaka (Figures 3-6, Table1).

># By changing the values of length and width we can analyze different sized of sidewalks (Figures 7-9).

> restart;

> k:=sum((m/c)^n/factorial(n),n=0..(s-1)):

> L:=8: W:=3: c1:=1.55*L*W: c:=ceil(c1): s:=(W-1.07)/0.8:cf:=c-1:K:=2*c:

> for m from 1 to K by 1 do f[m]:=evalf((1+((m/c)^s)/(s*(1-m/(s*c))*(m/c)^s+s*factorial(s)*(1-m/(s*c))^2*k))^(-1)) od:

> V[f]:=1.20:lambda:=w:

> for cf from 1 to cf by 1 do mf[cf]:=evalf(product(f[i],i=1..cf)): d[cf]:=evalf(factorial(cf)*mf[cf]): s[cf]:=((lambda*L/V[f])^cf)/d[cf]od:

> c:=ceil(c1): cf:=c-1:

> ss1:=sum(s[i],i=1..cf):

> c:=ceil(c1):K:=2*c:

> for i from c to K by 1 do mff[i]:=evalf(product(f[j],j=1..i)): df[i]:=evalf(factorial(c)*c^(i-c)*mff[i]): sf[i]:=((lambda*L/V[f])^i)/df[i]od:

> ss2:=sum(sf[x],x=c..K):

> P[0]:=(1+ss1+ss2)^(-1):

> c:=ceil(c1): cf:=c-1:

> for cf from 1 to cf by 1 do P[cf]:=evalf(((lambda*L/V[f])^cf)/(factorial(cf)*mf[cf])*P[0])od:

> c:=ceil(c1): cf:=c-1:EN1:=sum(r*P[r],r=1..cf):

> for i from c to K by 1 do P[i]:=evalf(((lambda*L/V[f])^i)/(factorial(c)*c^(i-c)*mff[i])*P[0])od:

> c:=ceil(c1): K:=2*c: EN2:=sum(t*P[t],t=c..K):

> EN:=EN1+EN2:

> c:=ceil(c1): K:=2*c:theta:=lambda*(1-P[K]):

> ET:=EN/theta:

> c:=ceil(c1): K:=2*c: Q:=sum((t-c)*P[t],t=(c+1)..K):

> with(plots):

> plot([P[K]],w=0..9,labels=["Arrival Rate(ped/sec)","The Probability of Balking (P[K])"],labeldirections=['horizontal','vertical'],thickness=2,axes=box, color=black, font=[COURIER,BOLD,12], legend=["Length=8m,Width=3m, vf=1.2"], legendstyle=[location=top]):

> plot([Q],w=0..9,labels=["Arrival Rate(ped/sec)","No. of Pedestrains in Queue (ped)"],labeldirections=['horizontal','vertical'],thickness=2,axes=box, color=black, font=[COURIER,BOLD,12], legend=["Length=8m,Width=3m, vf=1.2"], legendstyle=[location=top]):

> plot([ET],w=0..9,labels=["Arrival Rate(ped/sec)","Expected Amount of Time on the Facility(sec)"],labeldirections=['horizontal','vertical'],thickness=2 ,axes=box, color=black, font=[COURIER,BOLD,12], legend=["Length=8m,Width=3m, vf=1.2"], legendstyle=[location=top]):

> plot([theta],w=0..9,labels=["Arrival Rate(ped/sec)","Throughput(ped/sec)"],labeldirections=['horizontal','vertical'],thickness=2,axes=box, color=black, font=[COURIER,BOLD,12], legend=["Length=8m,Width=3m, vf=1.2"], legendstyle=[location=top]):

>

> #plot([EN],w=0..9,labels=["Arrival Rate","No of Pedestrains on the Facility"],labeldirections=['horizontal','vertical'],thickness=2,title="Expected Number of Pedestrians on the Facility for different Arrival Rates" ,axes=box, color=black, font=[COURIER,BOLD,12], titlefont=[TIMES,ROMAN,12], legend=["Length=8m,Width=3m, vf=1.2"], legendstyle=[location=top]);

># Following codes were used in R to analyze the effect of width on performances ( theta) in Figure 10 and Table 2.

getParam <- function(w=2.67,l=8,lambda=6,vf=1.2)

{

c<-ceiling(1.55*l*w)

s<-(w-1.07)/0.8

s1<-s-1

c1<-c-1

c2<-c+1

k<-2*c

es<-l/vf

m<-seq(1,k)

m_c<-m-c

mc<-m/c

ksum<-0

for(i in 0:s1){ksum<-ksum+mc^i/factorial(i)}

f<-1/(1+mc^s/(s*(1-mc/s)*mc^s+s*factorial(s)*(1-mc/s)^2*ksum))

d<-cumprod(f)

d[1:c1]<-factorial(m[1:c1])*d[1:c1]

d[c:k]<-factorial(c)*c^(m[c:k]-c)*d[c:k]

d<-(lambda*es)^m/d

p0<-1/(1+sum(d))

p<-p0*d

pb<-p[k]

theta<-lambda*(1-pb)

et<-sum(m*p)/theta

eq<-sum(m_c[c2:k]*p[c2:k])

rsl<-c(W=w,L=l,Lambda=lambda,Vf=vf,Pb=pb,EQ=eq,ET=et,Theta=theta)

return(rsl)

}

#replace .1 in w below by .01 to get more points

w<-seq(2.68,5,.1)

n<-length(w)

rslt<-matrix(rep(NA,n*8),n,8)

t<-rep(NA,n)

for(i in 1:n){x<-getParam(w[i],l=8,lambda=6,vf=1.2);for(j in 1:8){rslt[i,j]<-x[j]};t[i]<-x[8]}

plot(w,t,type="l",xlab="Width (m)",ylab="Throughput (ped/sec)")

nm<-c("W", "L", "Lambda", "Vf", "Pb", "EQ", "ET", "Theta")

rslt1<-t(rslt)

row.names(rslt1)<-nm

rslt<-t(rslt1)

rslt

># Result from the R was then used in Maple to draw the Figure 10

> restart;

> with(plottools);

> with(plots);

> pointplot([[2.68, 0.6521238e-1], [2.78, .70839877], [2.88, 1.31854096], [2.98, 1.88582812], [3.08, 2.47566693], [3.18, 2.97522609], [3.28, 3.44411060], [3.38, 3.89418520], [3.48, 5.06157732], [3.58, 5.66616312], [3.68, 5.93579200], [3.78, 5.99051611], [3.88, 5.99962618], [3.98, 5.99994990], [4.08, 5.99999306], [4.18, 5.99999901], [4.28, 5.99999998], [4.38, 6.00000000], [4.48, 6.00000000], [4.58, 6.00000000], [4.68, 6.00000000], [4.78, 6.00000000], [4.88, 6.00000000], [4.98, 6.00000000], [8, 6.00000000]], style = line, labels = ["Width (m)", "Throughput(ped/sec)"], labeldirections = ['horizontal', 'vertical'], thickness = 2, axes = box, color = black, font = [COURIER, BOLD, 12], legend = "Length=8 m,Width=3 m, vf=1.2", legendstyle = [location = top]);

># Following codes were used in Maple to analyze the effect of length on performances (P[K]; Q; ET; theta) in Tables 3 and 4.

># By changing the values of length we can analyze different sized of sidewalks

> restart;

> k:=sum((m/c)^n/factorial(n),n=0..(s-1)):

> L:=8: W:=3: c1:=1.55*L*W: c:=ceil(c1): s:=(W-1.07)/0.8:cf:=c-1:K:=2*c:

> for m from 1 to K by 1 do f[m]:=evalf((1+((m/c)^s)/(s*(1-m/(s*c))*(m/c)^s+s*factorial(s)*(1-m/(s*c))^2*k))^(-1)) od:

> V[f]:=1.20:lambda:=6:

> for cf from 1 to cf by 1 do mf[cf]:=evalf(product(f[i],i=1..cf)): d[cf]:=evalf(factorial(cf)*mf[cf]): s[cf]:=((lambda*L/V[f])^cf)/d[cf]od:

> c:=ceil(c1): cf:=c-1:

> ss1:=sum(s[i],i=1..cf):

> c:=ceil(c1):K:=2*c:

> for i from c to K by 1 do mff[i]:=evalf(product(f[j],j=1..i)): df[i]:=evalf(factorial(c)*c^(i-c)*mff[i]): sf[i]:=((lambda*L/V[f])^i)/df[i]od:

> ss2:=sum(sf[x],x=c..K):

> P[0]:=(1+ss1+ss2)^(-1):

> c:=ceil(c1): cf:=c-1:

> for cf from 1 to cf by 1 do P[cf]:=evalf(((lambda*L/V[f])^cf)/(factorial(cf)*mf[cf])*P[0])od:

> c:=ceil(c1): cf:=c-1:EN1:=sum(r*P[r],r=1..cf):

> for i from c to K by 1 do P[i]:=evalf(((lambda*L/V[f])^i)/(factorial(c)*c^(i-c)*mff[i])*P[0])od:

> c:=ceil(c1): K:=2*c: EN2:=sum(t*P[t],t=c..K):

> EN:=EN1+EN2:

> c:=ceil(c1): K:=2*c:theta:=lambda*(1-P[K]):

> ET:=EN/theta:

> c:=ceil(c1): K:=2*c: Q:=sum((t-c)*P[t],t=(c+1)..K):

> P[K];

> Q;

> ET;

> theta;

># Following codes were used in Maple for Figures 11-12 and Tables 5-6.

> restart;

> k:=sum((m/c)^n/factorial(n),n=0..(s-1)):

> L:=10: W:=2.8: c1:=1.55*L*W: c:=ceil(c1): s:=(W-1.07)/0.8:cf:=c-1:K:=2*c:

> for m from 1 to K by 1 do f[m]:=evalf((1+((m/c)^s)/(s*(1-m/(s*c))*(m/c)^s+s*factorial(s)*(1-m/(s*c))^2*k))^(-1)) od:

> V[f]:=w:lambda:=4:

> for cf from 1 to cf by 1 do mf[cf]:=evalf(product(f[i],i=1..cf)): d[cf]:=evalf(factorial(cf)*mf[cf]): s[cf]:=((lambda*L/V[f])^cf)/d[cf]od:

> c:=ceil(c1): cf:=c-1:

> ss1:=sum(s[i],i=1..cf):

> c:=ceil(c1):K:=2*c:

> for i from c to K by 1 do mff[i]:=evalf(product(f[j],j=1..i)): df[i]:=evalf(factorial(c)*c^(i-c)*mff[i]): sf[i]:=((lambda*L/V[f])^i)/df[i]od:

> ss2:=sum(sf[x],x=c..K):

> P[0]:=(1+ss1+ss2)^(-1):

> c:=ceil(c1): cf:=c-1:

> for cf from 1 to cf by 1 do P[cf]:=evalf(((lambda*L/V[f])^cf)/(factorial(cf)*mf[cf])*P[0])od:

> c:=ceil(c1): cf:=c-1:EN1:=sum(r*P[r],r=1..cf):

> for i from c to K by 1 do P[i]:=evalf(((lambda*L/V[f])^i)/(factorial(c)*c^(i-c)*mff[i])*P[0])od:

> c:=ceil(c1): K:=2*c: EN2:=sum(t*P[t],t=c..K):

> EN:=EN1+EN2:

> c:=ceil(c1): K:=2*c:theta:=lambda*(1-P[K]):

> ET:=EN/theta:

> c:=ceil(c1): K:=2*c: Q:=sum((t-c)*P[t],t=(c+1)..K):

> with(plots):

> plot([P[K]],w=0.1..2,labels=["Pedestrian Free Flow Speed (m/sec)","The Probability of Balking (P[K])"],labeldirections=['horizontal','vertical'],thickness=2,axes=box, color=black, font=[COURIER,BOLD,12], legend=["Length=10m,Width=2.8m, lambda=4ped/sec"], legendstyle=[location=top]);
